# Development of a YOLO-guided automated (microplastic) particle analysis workflow

**DOI:** 10.1039/d6ay00142d

**Published:** 2026-09-04

**Authors:** Junhao Xie, Aoife Gowen, Jun-Li Xu

**Affiliations:** a School of Biosystems and Food Engineering, University College Dublin Belfield Dublin 4 Ireland junhao.xie@ucdconnect.ie +353 (0) 87 231 5465

## Abstract

Microplastics (MPs) are an emerging pollutant of global concern, creating an urgent need for rapid and accurate monitoring workflows. Deep learning-based computer vision has demonstrated strong performance in finding particles in microscopy images, but its use as a front-end module for automated IR/Raman microscope-based MP analysis remains insufficiently developed, particularly in workflows that convert image-level particle detection results into microscope-executable operations for particle morphological characterization and spectral acquisition planning. Here, we aim to address this gap. First, reference MPs were deposited on Anodisc filters and glass slides, from which 626 bright-field micrographs containing 7010 particles were collected. The dataset was randomly split into training, validation, and test sets in a 7 : 2 : 1 ratio, and an Ultralytics YOLO11 instance segmentation model was developed. At an intersection-over-union (IoU) threshold of 0.7, testing achieved precision, recall, and F1 scores of 0.86, 0.93, and 0.89, respectively, outperforming two benchmark methods: a threshold-based method (F1 = 0.25) and Mask R-CNN (F1 = 0.84). On another test set prepared from tap water (with a relatively clean background), the model achieved precision, recall, and F1 scores of 0.78, 0.90, and 0.83 at IoU = 0.7. However, particle detection performance decreased with dirtier image backgrounds, as shown by testing on the sample prepared from commercial salt. We addressed key technical challenges required for end-to-end automation, including per-field autofocus, recovering true microscope coordinates from YOLO style outputs, converting detections into particle descriptors for downstream characterization, and programmatic microscope control for image collection and spectrum acquisition planning. Full implementation, code, and well annotated data are released openly, enabling adoption and extension of this workflow for broader MP monitoring applications.

## Abstract 2

A YOLO-guided workflow enables rapid and accurate particle detection, morphological characterization and spectral-acquisition planning for automated spectroscopic analysis of microplastics.

## Graphical abstract

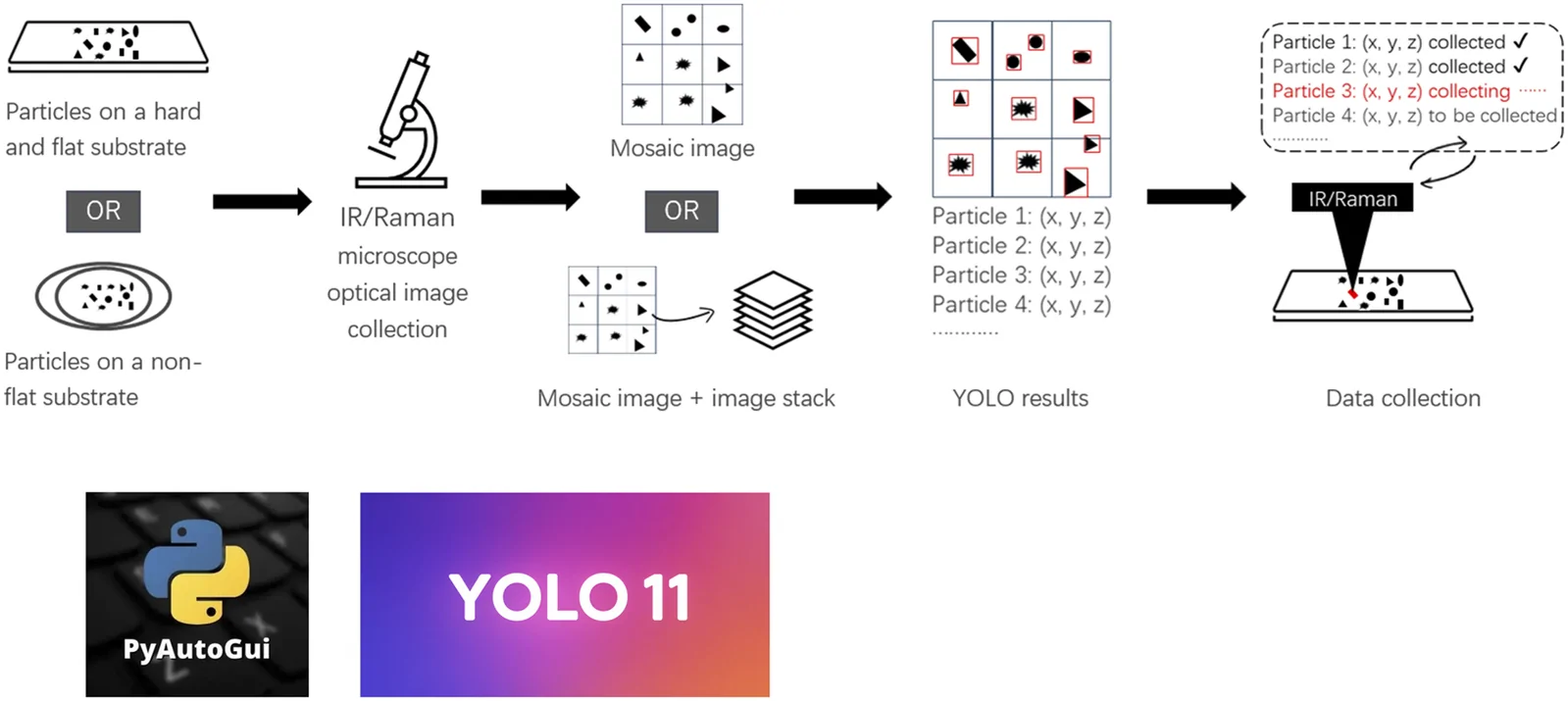

## 1 Introduction

Microplastics (MPs) are commonly defined as plastic fragments and fibers with a maximum dimension below 5 mm. They originate from a wide range of sources, including tire wear, shedding of microfibers from textiles during use and laundering, microbeads in personal care products, secondary fragmentation of larger plastic items under ultraviolet irradiation, mechanical abrasion, thermo-oxidative aging, and so on.^1^ A growing body of evidence has documented their pervasive presence across marine, freshwater, soil, sediment, air, foodstuffs and biological samples. Forecasting models have indicated that MP pollution loads are expected to double by 2040 (ref. 2). The ubiquity of MPs and their associated ecological and health risks have attracted sustained attention from governments, researchers, and the public, creating an urgent need for routine monitoring that both supports risk assessment and source attribution and provides objective baselines for evaluating policies and interventions.

Monitoring typically seeks to answer questions such as how many particles are present in a contaminated sample (*e.g.*, water, food, or biological tissues), what polymer types they are, and what their sizes and shapes are. A widely used analytical workflow begins with sample pretreatment to reduce organic and inorganic interferences through chemical or enzymatic digestion and density separation, followed by deposition of the remaining material onto a measurement-compatible substrate such as a filter for optical and spectroscopic microscopy. Particles are then located and described morphologically under a microscope, and infrared (IR) or Raman spectra are acquired from the identified objects to achieve chemical identification by comparison with reference libraries. Throughout the process, rigorous contamination control and quality assurance are essential.

At first, particle finding and morphological assessment relied on manual screening. When the substrate contains only a few particles, manual inspection can be adequate. As particle density increases, however, manual identification becomes slow and labor intensive, and selection bias is inevitably introduced. To address this issue, automated alternatives have been proposed. Traditional image processing offers an approach centered on threshold-based segmentation, with global thresholding, implemented either by manually assigning thresholds to the entire input image^3,4^ or by employing automatic thresholding methods.^5–7^ In practice, global thresholds are sensitive to heterogeneous illumination and background inhomogeneity. A single threshold behaves inconsistently across different regions of the same image, leading to false positives in some areas and missed detections in others. Variation in particle intensities further prevents one threshold from handling both bright and dim targets. To address these issues, adaptive thresholding has been introduced.^8^ The image is first converted to grayscale, and then a threshold is computed within a local neighborhood based on the distribution of nearby pixels, so each decision is governed primarily by local context. A light Gaussian blur is often applied beforehand to suppress background noise and to stabilize subsequent processing. Compared with global thresholding, results on specific datasets have shown that adaptive thresholding yielded significant improvements in true positive detection rates (compared with *Otsu* thresholding).^8^

Within threshold-based automatic particle finding and segmentation, agglomeration poses an additional difficulty. If touching particles are segmented as a single region, counts and morphological measurements are biased. The watershed algorithm has been used for de-agglomeration,^9,10^ but in samples containing fibers it may over-segment a single fiber. An adaptive de-agglomeration strategy^11^ mitigates this risk by triggering watershed only for objects that satisfy predefined shape criteria, for example non-fibrous targets with clear concavity or multi-peak structure.

Some threshold-based pipelines additionally integrate instrument control of spectroscopic microscopes to automatically acquire spectra from the detected particles, which improves analytical efficiency.^10,11^

In practice, threshold-based segmentation is typically applied to images where particle-background contrast is high, and background noise is low. Imaging techniques that deliver high particle-to-background contrast with limited noise include dark-field imaging, which requires a microscope configured for dark-field illumination;^12^ fluorescence imaging, which requires dyes such as Nile Red^6^ and a fluorescence microscope; scanning electron microscopy (SEM) imaging; and single-wavenumber IR imaging, where the background is non-absorbing or only weakly absorbing at a specific wavenumber (*e.g.*, 1800 cm^−1^), whereas the particles exhibit comparatively strong absorption. The Agilent 8700 laser direct infrared (LDIR) system exemplifies the last approach and relies on a tunable IR source, for example a quantum cascade laser (QCL).

Clearly, threshold-based automation depends on specialized instruments, auxiliary hardware, or reagents. Not all MP researchers have ready access to these resources, so the practical reach of such methods is limited. Similarly, automated approaches based on chemical imaging also rely heavily on dedicated platforms. For example, IR chemical imaging typically requires line-array detectors or focal-plane array (FPA) detectors; laboratories without line-array or FPA systems will find it difficult to adopt the corresponding workflows (*e.g.*, if only a single point IR detector is available, the imaging speed is extremely slow), which constrains their general applicability.

Just as threshold-based approaches have been applied to automatically locating and characterizing particles in microscopy images, deep learning-based computer vision (CV) methods (semantic and instance segmentation) have also been used to automate this task. Unlike thresholding, which often favors high-contrast imaging, these deep learning methods could operate under more relaxed conditions: they work not only on images with strong particle–background contrast but also commonly on low-contrast images (*e.g.*, bright-field images), and they exhibit strong robustness to background noise. A variety of deep learning-based CV models, for example U-Net,^13–17^ Mask R-CNN,^14,18–21^ and the YOLO family,^15,21^ have achieved excellent particle and fiber segmentation performance on microscopy images. The literature includes feasibility demonstrations on relatively clean backgrounds as well as high-quality segmentation on real-world environmental samples with visually noticeable background noise,^15,20,21^ further demonstrating the practical effectiveness of this paradigm. Some studies also directly benchmarked against thresholding and reported superior performance; for example, Park *et al.*^22^ proposed MP-Net, a deep learning-based segmentation model that overcame the overestimation problem of threshold-based MP-VAT^6^ and MP-VAT 2.0 (ref. 7) on the same fluorescence micrographs.

Despite the strong performance of deep learning in particle segmentation, to our knowledge, no automated MP analysis workflow has been developed that integrates deep learning-based CV with IR/Raman microscopes in a way that enables particles to be localized, their precise positions in the microscope XYZ coordinate system to be automatically transferred to the microscope-control software for spectral-acquisition planning, and particles to be segmented for automated particle-level morphological characterization, including the extraction of particle area, circularity, maximum Feret diameter, and minimum Feret diameter. Hence, the aim of this work was to develop such an integrated workflow. We selected YOLO11 as the core segmentation model. Earlier YOLO-based implementations for particle analysis in micrographs have mainly relied on YOLOv5 (ref. 15) and YOLOv8 (ref. 21 and 23). As a more recent development in the YOLO family, YOLO11 has shown improved performance on the COCO benchmark.^24^ We therefore adopted YOLO11 in this study (also, comparative experiments showed that, for the present task, YOLO11 achieved slightly better overall performance than those earlier YOLO versions; please see Section 3.1). PyAutoGUI was used as a software-level automation layer to coordinate the spectroscopic microscope and the YOLO model within the workflow, enabling the automated execution of the overall process. The developed workflow is applicable to IR/Raman microscopes equipped with a single-element detector, a microscope camera, a motorized sample stage, and software capable of controlling the motorized stage (which are common features of modern IR/Raman microscopes). We also released a well-annotated dataset comprising 10 239 particles across 1985 images and demonstrated that lightweight fine-tuning can substantially improve generalization. Finally, the workflow is model agnostic, since it only requires standard instance-level outputs such as particle locations and masks, and predictions exported in different formats can be mapped *via* a lightweight adapter to the unified representation used for coordinate mapping and automated spectral acquisition planning.

## 2 Materials and methods

### 2.1 Sample preparation

Four types of pristine MPs with different particle morphologies were used as reference particles in this study. These included polyamide 6 (nylon) fragments (15–20 µm in maximum dimension; irregular in shape; white in color; Goodfellow, UK), polyethylene terephthalate (PET) fragments (30–300 µm in maximum dimension; irregular in shape; white in color; Goodfellow, UK), polypropylene (PP) fragments (10–90 µm in maximum dimension; irregular in shape; white in color; Goonvean Fibres, UK), and polystyrene (PS) microspheres (45 µm in diameter; white in color; perfectly spherical; Polysciences, USA). These reference MPs were prepared on both Anodisc aluminum oxide filters and glass slides for subsequent image collection.

For the filter samples, 0.05 mg of MP powder was first spiked into 100 mL deionized water and then enriched on Anodisc aluminum oxide filters (0.2 µm pore size and 25 mm in diameter; Anodisc, Whatman, UK) by vacuum filtration. The prepared filters were air-dried in a fume hood before subsequent experimental steps.

For the glass-slide samples, 0.05 mg of dry MP powder was directly transferred onto clean glass slides using a clean metal spatula, gently spread over the slide surface until particle aggregation was no longer visible to the naked eye, and then used directly for optical image acquisition.

The final prepared reference samples include nylon on filter, nylon on glass slide, PET on filter, PET on glass slide, PP on filter, PP on glass slide, and PS on glass slide.

In addition, two environmental samples were prepared. The first sample was from tap water. Specifically, 100 mL of tap water was taken from a kitchenette in the Science South building at University College Dublin, Ireland. The water was collected in a clean glass beaker, immediately covered with aluminum foil, transported to the laboratory (in the same building), and filtered onto a silicone membrane in a fume hood. After the beaker had been emptied, its inner wall was rinsed with deionized water, and the rinse water was passed through the same membrane to recover any particles remaining on the beaker wall. The membrane has square pores of about 5 µm × 5 µm, and the filter diameter is 20 mm. The filter was air-dried in a fume hood before proceeding with subsequent experimental steps. The second sample was a ground-salt sample prepared following the salt-grinding procedure described by Yang *et al.*^25^ Briefly, 50 g of rock salt from a commercial salt grinder product was ground using the product's own grinder head and collected in a clean borosilicate glass beaker. After grinding, 30 mL of 30% (w/w) hydrogen peroxide was added to remove organic impurities. The mixture was heated at 75 °C and stirred at 300 rpm for 45 min, then cooled to room temperature. Subsequently, 250 mL of deionized water was added, followed by stirring at 300 rpm for 30 min. The beaker was then kept covered and left undisturbed for 24 h. The supernatant was collected for filtration, and the resulting filter was air-dried before subsequent analysis.

Throughout the sample preparation process, several measures were taken to reduce contamination. All containers and tools that came into direct contact with the samples were made of glass or metal, including glass beakers, glass slides, glass Petri dishes, the filtration apparatus, and metal spatulas; these items were cleaned before use and thoroughly rinsed with deionized water. Powder-free nitrile gloves were worn during sample handling. The tap-water sample was covered with aluminum foil immediately after collection and transported to the laboratory; filters and glass slides were placed in covered glass Petri dishes during transfer and storage. Filtration, drying, and related sample-handling steps were performed in a fume hood.

### 2.2 Instrumentation and image collection

The microscope used herein was a mIRage IR microscope (Photothermal Spectroscopy Corp, Santa Barbara, California, USA). This microscope is equipped with a single-element detector, a motorized sample stage, a QCL IR source (spectral range: 1801 cm^−1^–769 cm^−1^), a microscope camera, a low-magnification 10× objective and a high-magnification 40× objective. PTIR Studio (Version 4.4.8208, Photothermal Spectroscopy Corp, Santa Barbara, California, USA) was the software used to control the mIRage IR microscope. This software allows users to switch objectives, move the motorized stage, collect IR spectra from designated coordinates (*x*, *y*, *z*), acquire optical images, and so on.

Using the mIRage IR microscope, optical images of the prepared samples were captured. After filtering out images displaying severe particle aggregation (as ground truth annotation for images with severely aggregated particles is impossible), a total of 2001 images were retained. Subsequently, these retained images were annotated, during which it was ensured that the annotation boundaries closely followed the true edges of each particle, and all particles were labelled as “target” (hence, the number of classes is one for this dataset). Ultimately, a total of 10 502 instances/particles were identified from the 2001 optical images and well annotated. Table 1 summarizes the number of images and annotated particles for each type–substrate combination.

**Table 1:** Number of images and annotated particles for each type–substrate combination. Ref = reference

Sample	Number of images	Number of particles
Ref nylon on Anodisc filter	140	2551
Ref nylon on glass slide	69	839
Ref PP on Anodisc filter	103	1238
Ref PP on glass slide	76	682
Ref PET on Anodisc filter	98	1069
Ref PET on glass slide	110	518
Ref PS on glass slide	30	113
Tap-water filtrate on silicone membrane	1359	3229
Commercial salt filtrate on Anodisc filter	16	263
Sum	2001	10 502

To improve annotation efficiency, a model-assisted pre-annotation strategy was used. Specifically, a preliminary YOLO-seg model was first trained on a small subset of manually annotated images and then applied to the remaining unannotated images to generate rough segmentation masks. These model-generated masks were used only as editable initial annotations and were not directly accepted as ground-truth annotations. Subsequently, all masks were checked and refined by experts using the Open Data Annotation Platform (https://app.cvat.ai/). The final annotation criterion was that the segmentation boundaries should closely follow the visually identifiable edges of each particle. This model-assisted strategy substantially reduced the time and effort required for annotation. Because the final annotations were expert-refined rather than unmodified preliminary model predictions, this strategy was used only to improve annotation efficiency and did not directly introduce preliminary-model errors into the final ground-truth annotations or the subsequent model-error calculation.

### 2.3 Training a YOLO segmentation model

YOLO is an architecture proposed by Redmon *et al.*,^26^ extensively applied in CV tasks due to its real-time, high-accuracy object detection. Since its introduction, YOLO has undergone significant improvements, giving rise to multiple versions such as YOLOv5, YOLOv7, and YOLO11. Its functionality has expanded from the initial focus on object detection to include instance segmentation, pose/keypoint estimation, oriented object detection, and image classification. Currently, the latest version is YOLO11, released by Ultralytics. YOLO11 provides networks of different scales, namely YOLO11n, YOLO11s, YOLO11m, YOLO11l, and YOLO11x, each with increasing depth leading to improved performance, although at the cost of greater computational demand.

In this project, we worked with YOLO11l and trained an instance segmentation model, *i.e.*, a YOLO-seg model. The 626 images prepared from reference MPs were randomly divided into training, validation, and test sets using a 7 : 2 : 1 ratio. The training set was used to learn the model parameters, the validation set was used for early stopping, and the test set was reserved for the final unbiased evaluation.

The images prepared from tap water and commercial salt represent a real-world environmental test set to assess generalization.

Training was initialized from Ultralytics' YOLO11l pretrained weights and targeted 1000 epochs, with early stopping (patience = 50) enabled to terminate training if the validation metric failed to improve for 50 consecutive epochs. Although many hyperparameters can be specified, we kept the defaults except for a few key ones. For particle instance segmentation in microscopy images, we primarily adjusted imgsz (input resolution) and mask_ratio (mask sampling/encoding ratio): a larger imgsz preserves small particles and fine boundaries and improves edge adherence, so we increased it from the default 640 to 672; if mask_ratio is too large, for example the default value of 4, masks exhibit insufficient edge detail with visibly jagged boundaries, so we set it to 1 to preserve mask detail, analogous to anti-aliasing. For a complete list of all hyperparameters specified at initialization, please refer to the model training code.py and default parameters.yaml submitted with the manuscript; the access link is provided in Section 5.

The training was performed on the Google Colab platform using an NVIDIA T4 GPU. The training process was terminated by the early-stopping mechanism at epoch 185, and the total elapsed training time from initialization to termination was approximately 5.4 hours.

### 2.4 Developing an automated particle detection workflow

A Python script was developed to automate the following processes: optical image collection, YOLO-seg inference, generation of target coordinates for subsequent spectral acquisition from the particles identified by the model, and morphological characterization of the detected particles.

The “automation” part was mainly enabled by controlling the microscope through the combined use of PTIR Studio and PyAutoGUI. PyAutoGUI is a Python module designed for automating interactions with a computer's graphical user interface (GUI). It offers a set of functions that can simulate mouse and keyboard actions, such as clicking, dragging, and typing. This module is useful for automating repetitive tasks, GUI testing, data entry, and various other automation purposes.

During the collection of optical images, a region of interest (ROI) and a good focal distance *z* (the distance between the microscope camera and the sample stage at which the field of view of the camera is optimal, or clear) are initially designated by the operator. Subsequently, the Python program moves the motorized stage and orders the microscope camera to acquire optical images (the size of each image is 639 µm in width × 480 µm in height, corresponding to the size of the field of view of the camera) from the ROI in the way depicted in Fig. S1 (in other words, multiple small images are collected to form a large mosaic image to cover the designated ROI).

The microscope used in this study is equipped with two objectives, a 10× objective and a 40× objective. With the 10× objective, the camera captures a field of view measuring 639 µm in width and 480 µm in height at the sample plane. With the 40× objective, the field of view is 166 µm × 124 µm. All procedures described in Section 2.4 are based on the configuration with an effective field of view of 639 µm × 480 µm at the sample plane.

During the horizontal movement of the motorized stage for image collection, refocusing (updating *z*) is not needed if the substrate (where MPs are deposited) used is hard and flat, such as a glass slide. However, when using a substrate that is not naturally flat, such as an aluminum oxide filter, defocusing is almost inevitable as the motorized stage moves, particularly with a large ROI. We tested a filter holder to flatten the filter surface, but this did not solve the problem in our microscope because the high-NA objective used for image acquisition, while providing high spatial resolution, has a very shallow depth of field, so even very small residual warping or tilt of the filter still leads to defocus during stage movement.

To mitigate this defocusing, the Python script integrates a per-field automated focus routine to determine the optimal focal distance *ẑ* for each field of view. Specifically, within the camera's single-field area of 639 µm × 480 µm, images are acquired as a *z* stack by stepping along *Z* (stepping vertically) at a fixed interval. Starting from an initial reference focus *z*, an image is first acquired at this plane. The sample stage is then moved upward by *n* steps with a step size of *k* µm, with one image acquired at each step. After the stage is returned to the reference focus, it is moved downward by a further *n* steps with the same step size, again with one image acquired at each step. In total, a stack of 2*n* + 1 images is obtained, one of which is in the best focus. The trained YOLO-seg model is then used to automatically identify the sharpest frame in the stack (explained in the next paragraph), and the focal distance value corresponding to that frame is taken as the optimal focal distance *ẑ* for that field of view.

**Fig. 1:**
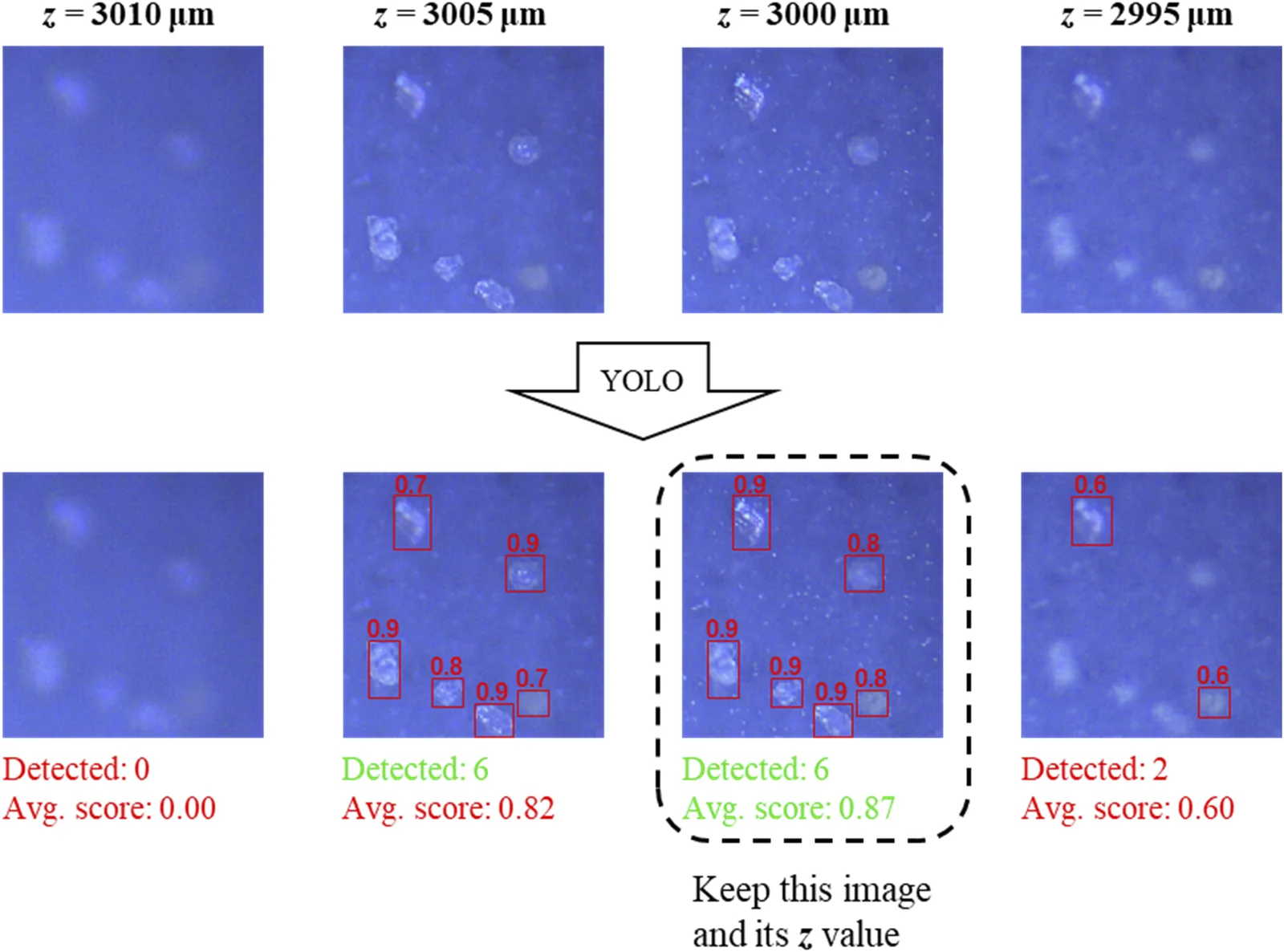
YOLO-seg-assisted selection of the sharpest frame and the optimal focal distance from a *z* stack, illustrated using a 4-frame *z* stack.

To automatically select the best focal plane from each *z* stack, the trained YOLO-seg model is run on every frame of the stack and two metrics are recorded: the number of detected objects and the mean confidence score. The decision rule is as follows: if a frame contains the highest count of YOLO detections and also has the highest average confidence score, it is considered the clearest image (Fig. 1) and is retained, while the other frames are discarded. The corresponding focal distance value is then taken as the optimal focal distance *ẑ* for the region shown in that image.

At this stage, optical images have been collected for the ROI, regardless of the substrate used. In the automated detection workflow developed in this study, the next step is to identify particles in these optical images and to acquire spectra from the identified particles. To this end, the true three-dimensional microscope coordinates (*x*, *y*, *z*) of a representative point on each identified particle must be obtained. *z* is actually a known value at this stage, as described above: when the substrate is flat, a single, ROI-wide *z* (the value specified by the operator at the beginning) can be used; when the substrate is not flat, a per-field autofocus routine is required to determine the local *z*. Therefore, this section focuses on how to obtain the true *x* and *y*. Using a single camera field of view (639 µm × 480 µm) as an example, we show how to obtain the true microscope coordinates (*x*, *y*) of a representative point on each detected particle.

For the *i*-th segmented region within this field, the YOLO-seg outputs can be used to compute normalized coordinates (*x*_c_, *y*_c_) for a point located within that segmented region ((*x*_c_, *y*_c_) is defined in this work as the center of the maximum inscribed circle; see Section 3.2 for details on why the center of the maximum inscribed circle was chosen and how *x*_c_ and *y*_c_ are computed). Because these coordinates are expressed in the YOLO coordinate system, they must be converted into the physical coordinates on the sample stage. We define a Cartesian coordinate system on the 639 µm × 480 µm field whose *X* and *Y* axes intersect at the image center. Let this intersection have physical coordinates (*x*_0_, *y*_0_), corresponding to the true projected coordinates of the center of the current microscope field on the sample stage; in practice, (*x*_0_, *y*_0_) are known from the microscope control software. Fig. S2 compares the YOLO coordinate system (panel a) with this field-of-view-based coordinate system (panel b). By observation, mapping YOLO coordinates to the field-of-view coordinate system requires translation, scaling, and vertical flipping. Specifically, for any YOLO coordinate (*x*_p_, *y*_p_), its coordinates in the field-of-view coordinate system (*i.e.*, the true coordinates on the sample stage) are: (*x*_p_ * 639 µm − 319.5 µm + *x*_0_, *y*_p_ * −480 µm + 240 µm + *y*_0_). Accordingly, for the *i*-th segmented region within the 639 µm × 480 µm field, the in-plane components of the true spectral-acquisition point are *x*_c_ * 639 µm − 319.5 µm + *x*_0_, *y*_c_ * −480 µm + 240 µm + *y*_0_. Combined with the corresponding *z* for that region, these give the full physical coordinates of the particle's spectral-acquisition point in the microscope.

The above transformation has been implemented in the Python-based control workflow and is applied automatically to every field of view and every segmented region, yielding spectral-acquisition coordinates for all detected particles within a given ROI. Once these coordinates have been computed, they are fed to the microscope control software to enable fully automated spectral data collection for all particles detected in that ROI.

The YOLO-seg outputs are used not only to determine the spectral-acquisition coordinates of every detected particle, but also to compute each particle's area, circularity and its maximum and minimum Feret diameters. All of these quantities are calculated automatically by pre-implemented algorithms integrated into the same automated workflow. The corresponding calculation procedures, as well as the principles for converting YOLO-format outputs into real-world particle dimensions in micrometers, are detailed in Section 3.2.

The automated workflow described in this section has been deployed on the mIRage IR microscope to scan an Anodisc filter onto which MPs were deposited. A demo video is submitted with the manuscript; the access link is provided in Section 5.

In principle, the workflow is applicable to any IR or Raman microscopy system whose control software displays the optical microscope image in real time, provides a control panel for the motorized stage, accepts user input or programmatic calls for target XYZ coordinates, and allows single-point spectral acquisition to be triggered directly from the software interface.

The current implementation is tailored to our mIRage system, relying on PyAutoGUI to automate the graphical user interface. The procedure depends on interface details such as window layout, control positions and names, screen resolution and scaling, keyboard shortcuts, *etc.* These aspects vary substantially across microscope control software, and they also vary across computers and displays. Therefore, before applying the workflow to another microscope, or to another laboratory's mIRage installation, the GUI automation layer must be adapted by modifying the PyAutoGUI control code. We provide the complete code for the automated workflow (see Section 5).

If the microscope-control software provides open instrument-control interfaces, such as APIs or SDKs, allowing users to directly call functions for stage movement, autofocus, coordinate input, and spectral acquisition, a deeper level of software–hardware integration could be achieved. However, such interfaces are not universally available across commercial IR/Raman microscope systems. The PyAutoGUI-based control layer used in this study is a pragmatic implementation under the constraints of proprietary microscope-control software.

## 3 Results and discussion

### 3.1 Model performance

The initial training configuration was set for 1000 epochs with an early-stopping mechanism enabled (patience = 50). According to the training log, training stopped at epoch 185. Considering the early-stopping patience setting, the actual best-performing model was the one updated at the 135th epoch. The best YOLO-seg model was saved and used subsequently for further analysis. During training, a gradual decreasing and converging trend was observed for all losses, including box loss, segmentation loss, class loss, and distributed focal loss (Fig. S3). Concurrently, the performance metrics on the validation set, including precision, recall, mAP@0.5, and mAP@0.5 : 0.95, gradually increased and reached stable convergence (Fig. S3).

When the best-performing trained model was evaluated on the test set, the obtained metrics were mAP@0.5 = 0.96 and mAP@0.5 : 0.95 = 0.59. These results indicate that the model has achieved very high performance (mAP = 0.96) under the relatively lenient criterion (IoU ≥ 0.5, IoU is short for Intersection over Union). However, the performance slightly decreased (mAP = 0.59) under the stricter evaluation criterion (IoU range from 0.5 to 0.95).

At an IoU threshold of 0.7, the model achieved 608 true positives (TP), 101 false positives (FP), and 43 false negatives (FN) on the test set, yielding precision = 0.86, recall = 0.93, and F1-score = 0.89. These outcomes represent relatively ideal performance, as an IoU threshold of 0.7 imposes a relatively strict standard for segmentation overlap. The high degree of consistency between the ground truth contours and the YOLO-predicted segmentation contours on the test set is visualized in Fig. S4. The ground-truth masks were manually annotated by trained human experts and served as the reference standard. Hence, the reported TP, FP, FN, precision, recall, and F1-score quantify the degree of agreement between the YOLO-predicted contours and expert-derived annotations. The high recall and F1-score indicate that the model successfully recovered most expert-identified targets and achieved strong consistency with human expert annotation, although the remaining FPs and FNs show that the model did not perfectly reproduce expert judgement.

Early microscopy image-based studies used instance segmentation to quantify MP fibers, demonstrating that machine-learning-based segmentation methods can support the automated analysis of morphologically complex MPs.^18^ CV systems such as SMACC further demonstrated that MPs can be automatically counted and classified from digital images.^27^ Lorenzo-Navarro *et al.*^28^ applied a deep-learning workflow and reported a Jaccard index of 0.8 for particle segmentation and a classification accuracy of 98.11% for MP particles. Shi *et al.*^13^ applied U-Net- and MultiResUNet-based segmentation methods to scanning electron microscopy images of MPs and reported an average Jaccard index above 0.75, together with high shape-classification accuracy. For microscopy image-based segmentation tasks, Park *et al.*^22^ proposed MP-Net to segment fluorescence microscopy images of MPs isolated from clam samples and reported a mean F1-score of 0.74 and a mean IoU of 0.62. Huang *et al.*^20^ applied a Mask R-CNN-based approach to microscopy images and reported a segmentation F1-score of 0.76. More recently, Hao *et al.*^15^ developed a two-stage framework combining an improved YOLOv5 model for MP localization and an adaptive perceptual UNET 3+++ model for segmentation of adherent MPs in optical micrographs, reporting an mIoU of up to 96% and an average F1-score of 99.65%.

These previous studies demonstrate that machine-learning-based methods achieved strong performance in image-based MP analysis. However, their reported results cannot be directly compared with ours unless the different methods are evaluated on the same dataset using the same evaluation criteria. Therefore, in Section 3.1.1, we further compared our YOLO-seg model with two representative methods on the same dataset to obtain a fairer assessment of model performance.

We changed the illumination conditions of the test-set images by applying different illumination perturbations, including a cool-white color tone, edge vignetting, a local illumination hotspot, and uneven field illumination. Then, these altered images were used to test the model. The results showed that the YOLO-seg model maintained segmentation performance close to that obtained on the original test set under these conditions (Fig. S5). Compared with the baseline mAP@0.5 and mAP@0.5 : 0.95 values of 0.959 and 0.592, respectively, the mAP@0.5 values under these illumination perturbations remained between 0.950 and 0.958, and the mAP@0.5 : 0.95 values remained between 0.581 and 0.592 (Table S1). This stability is attributable to the HSV color-space augmentation enabled by default during YOLO training, including random perturbations of image hue, saturation, and brightness; the brightness augmentation directly simulates image changes under different illumination intensities.^24^ Because the illumination conditions and light-source characteristics may differ among microscope systems, these results suggest that the model has a certain degree of cross-microscope-system generalization ability.

Although YOLO11 has shown better performance than earlier versions such as YOLOv5 and YOLOv8 on public datasets such as COCO,^24^ this advantage may not necessarily translate directly to small-object segmentation in microscopy images. Therefore, a small-scale comparative investigation into this was conducted. We compared YOLO11l-seg with YOLOv8l-seg and YOLOv5l-seg under the same data split, training settings, and evaluation procedure. The results are summarized in Table 2. Overall, YOLO11l-seg showed the most balanced performance. It gave the highest mAP@0.5, although the difference from YOLOv8l-seg and YOLOv5l-seg was small. It also achieved the highest F1 score at IoU = 0.7. In terms of inference speed, YOLO11l-seg was slightly slower than YOLOv5l-seg but faster than YOLOv8l-seg. However, YOLO11l-seg did not outperform the earlier versions in every metric; in particular, it achieved slightly lower mAP@0.5 : 0.95 in comparison. Therefore, these results should not be interpreted as showing an across-the-board superiority of YOLO11. Rather, they indicate that YOLO11l-seg provided the best overall balance among segmentation performance at the evaluation threshold used in this study, mask-level detection accuracy, and computational efficiency. These comparative results therefore support the selection of YOLO11 as the segmentation model here.

**Table 2:** Performance comparison of YOLO11l-seg, YOLOv8l-seg and YOLOv5l-seg on the same test set. All three models were evaluated using the same data split and evaluation procedure. Inference time was measured on an NVIDIA T4 GPU

Model	mAP@0.5	mAP@0.5 : 0.95	F1 (IoU = 0.7)	Inference time (ms per image)
YOLO11l-seg	0.959	0.592	0.894	42.94
YOLOv8l-seg	0.957	0.613	0.877	53.00
YOLOv5l-seg	0.956	0.658	0.829	41.30

#### 3.1.1 Comparison with existing particle finding methods

The primary objective of this study is to develop an automated MP analysis framework based on the YOLO-seg model. This framework is centered on the YOLO-seg model for particle finding and incorporates PyAutoGUI to enable automated operation of the entire process. The design concept is highly similar to that of TUM-ParticleTyper 2 developed by Jacob *et al.*,^11^ which also employs a particle detection algorithm at its core and uses PyAutoGUI for automated operation. Therefore, TUM-ParticleTyper 2 is selected as a benchmark method for comparative analysis in this study.

TUM-ParticleTyper 2 is an upgraded version of the original TUM-ParticleTyper.^8^ The second-generation software inherits the innovative adaptive thresholding approach from its predecessor, which has demonstrated up to a twofold improvement in performance compared to the conventionally used *Otsu* thresholding method. In addition, the software introduces a novel adaptive de-agglomeration algorithm. More details of TUM-ParticleTyper 2 are not elaborated here and can be found in the studies by Von Der Esch *et al.*^8^ and Jacob *et al.*^11^

For comparison, we evaluated the particle segmentation performance of both the YOLO-seg model and TUM-ParticleTyper 2 using our test dataset prepared from reference MPs. The quantitative results (based on an IoU threshold of 0.7) are shown in Table 3. For visual comparison, the segmentation contours generated by both methods, along with the ground truth contours, were overlaid on the test images (Fig. S4).

**Table 3:** Comparison of performance between our YOLO-seg model and TUM-ParticleTyper 2 on the test set, evaluated at IoU threshold = 0.7. TP = true positive, FN = false negative, FP = false positive, P = precision, and R = recall

Method	TP	FN	FP	P	R	F1
YOLO-seg	608	43	101	0.86	0.93	0.89
TUM-ParticleTyper 2	217	434	854	0.20	0.33	0.25

Overall, the YOLO-seg model showed strong performance across the evaluation metrics. The test set contains 651 manually annotated particles. The YOLO-seg model correctly identified 608 (608 TPs), missed 43 (43 FNs), and produced 101 FPs.

**Fig. 2:**
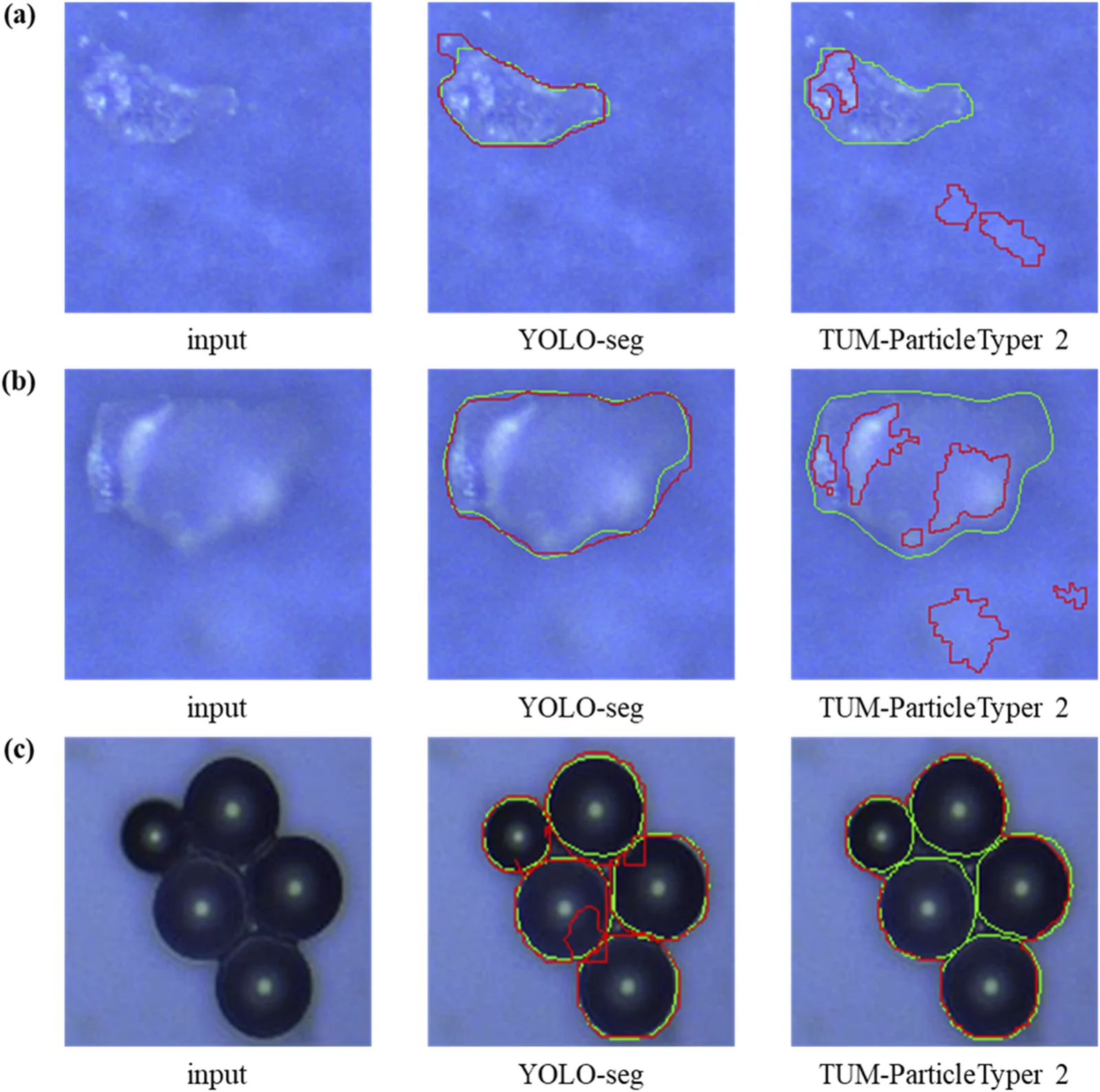
Segmentation contours produced by our YOLO-seg model and TUM-ParticleTyper 2 on two test images of particles on an Anodisc filter (a and b) and one test image of particles on a glass slide (c). Green: ground truth contours; red: segmentation contours generated by the YOLO-seg model or TUM-ParticleTyper 2. For visualized results across all test images, see Fig. S4.

TUM-ParticleTyper 2 achieved only 217 TPs, indicating that many ground truth particles were either not assigned a corresponding segmentation mask or were assigned masks that did not reach the required IoU threshold of 0.7 with the ground truth. Further analysis revealed that TUM-ParticleTyper 2 was quite effective at locating particles. When the IoU threshold was reduced to 0.01, TUM-ParticleTyper 2 achieved a TP count of 515, meaning that it was able to detect approximately 79% of all the particles. This suggests that the main limitation may have been related less to particle localization than to generating masks with sufficient overlap with the ground truth. Indeed, visualized results show that this benchmark method tended to produce one or more segmented regions on a single particle, but none of them individually covered more than 70% of the corresponding ground truth area (Fig. 2a and b). It also tended to generate a single segmentation region for particles closely clustered together (Fig. 2c). The former was likely caused by variations in brightness across different parts of a particle, with TUM-ParticleTyper 2 tending to detect only the brighter regions as particles. The latter might be because the de-agglomeration mechanism was not activated in such cases.

The low TP count led to a high FN count (since TP + FN = the total number of labeled particles here). Therefore, the main reason for the high FN value of the benchmark method was its low TP value.

**Fig. 3:**
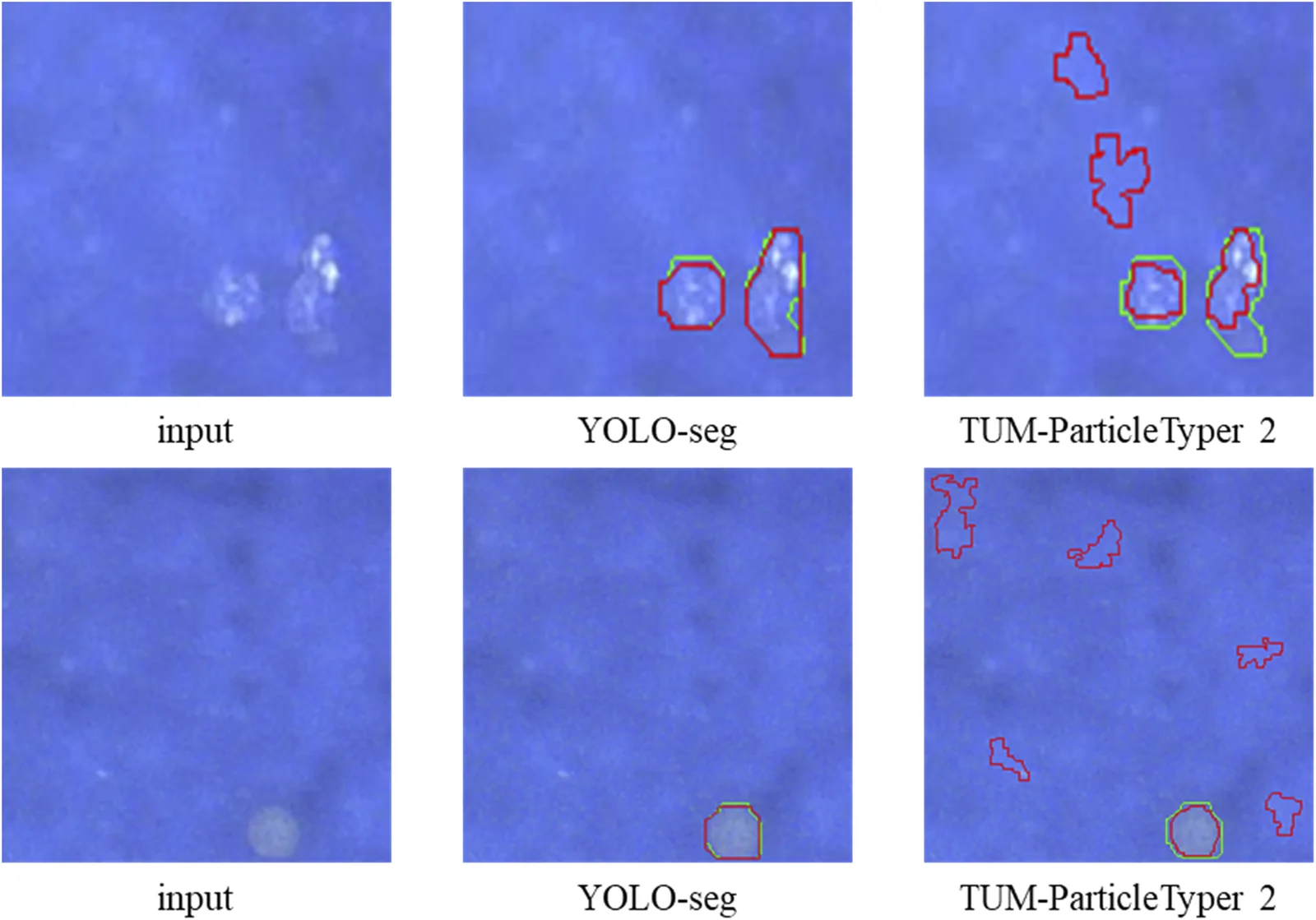
Segmentation contours produced by our YOLO-seg model and TUM-ParticleTyper 2 on two test images of particles on Anodisc filters. Green: ground truth contours; red: segmentation contours generated by the YOLO-seg model or TUM-ParticleTyper 2. For visualized results across all test images, see Fig. S4.

At an IoU threshold of 0.7, one of the main contributors to the high FP rate of TUM-ParticleTyper 2 was the generation of one or more segmentation regions on a single particle, none of which covered more than 70% of the particle's area. This is exemplified in Fig. 2a and b. Focusing solely on the particle region, Fig. 2a contributed one FN and one FP simultaneously, while Fig. 2b contributed one FN along with four FPs. Another major contributor was the misidentification of background regions as particles (Fig. 2a and b and 3). This suggests that the method might be sensitive to background noise.

Consequently, on the test set, the TP, FP, and FN counts obtained with TUM-ParticleTyper 2 were reflected in lower precision, recall, and F1-score. In comparison, the YOLO-seg model was far less affected by these issues, which explained its much stronger performance across these metrics.

Visual inspection of all results (Fig. S4) revealed that the performance gap between the two methods was most pronounced in images with low particle-to-background contrast. In such cases, TUM-ParticleTyper 2 produced more FNs and FPs, whereas the YOLO-seg model correctly identified most of the particles missed by TUM-ParticleTyper 2 and largely avoided misclassifying background regions as particles.

In our test set, images of particles on Anodisc filters corresponded to the low particle–background contrast category, whereas those on glass slides represented the high-contrast category. The performance metrics for the two methods on these two categories are presented in Table 4.

**Table 4:** Comparison of performance between our YOLO-seg model and TUM-ParticleTyper 2 on test images of particles on Anodisc filters and glass slides, evaluated at IoU threshold = 0.7. TP = true positive; FN = false negative; FP = false positive; P = precision; R = recall

Method	Substrate	TP	FN	FP	P	R	F1
YOLO-seg	Anodisc filter	414	30	79	0.84	0.93	0.88
YOLO-seg	Glass	194	13	22	0.90	0.94	0.92
TUM-ParticleTyper 2	Anodisc filter	80	364	828	0.09	0.18	0.12
TUM-ParticleTyper 2	Glass	137	70	26	0.84	0.66	0.74

It could be seen that TUM-ParticleTyper 2 was less well suited to images of particles on Anodisc filters than the YOLO-seg model, producing approximately fivefold fewer TPs and over ten times more FNs and FPs. In contrast, on images of particles on glass slides, its performance was much better, with only a small gap compared to the YOLO-seg model.

It should be emphasized that the reduced performance of TUM-ParticleTyper 2 on low-contrast particle–background images does not indicate an inherent limitation of the method. The authors of the software have made it very clear that TUM-ParticleTyper/TUM-ParticleTyper 2 is designed for dark-field, SEM, and fluorescence images, where particles are very well distinguished from the background.

**Fig. 4:**
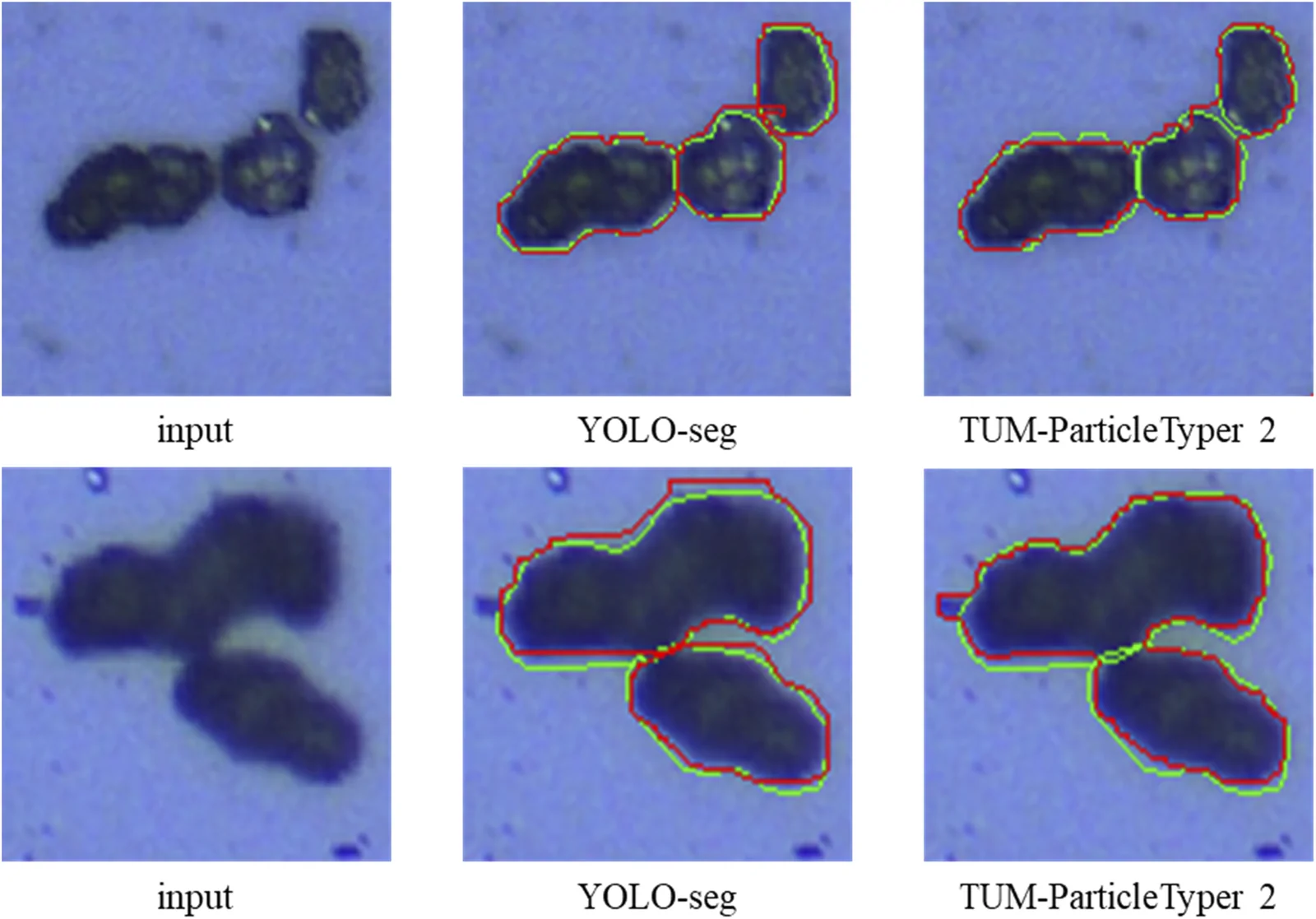
Segmentation contours produced by our YOLO-seg model and TUM-ParticleTyper 2 on two test images of particles on glass slides. Green: ground truth contours; red: segmentation contours generated by the YOLO-seg model or TUM-ParticleTyper 2. For visualized results across all test images of particles on glass slides, please see Fig. S4.

Focusing solely on images of particles on glass slides (high-contrast images), it could be seen that the YOLO-seg model and TUM-ParticleTyper 2 achieved comparable and high precision values. However, the recall differed substantially: the YOLO-seg model achieved 0.94 compared to 0.66 for TUM-ParticleTyper 2, indicating that the latter missed more particles. Visual inspection of these high-contrast test images revealed two main types of missed detections (Fig. S4). The first involves missing isolated, scattered particles whose contrast against the glass slide is lower than that of most other particles (while images of particles on glass slides are high contrast for the majority of particles, a subset still exhibits relatively low contrast with the background). The second involves limited separation of touching or clustered particles, for example, producing only a single segmentation for a cluster. In many cases, clusters consisting of two or more particles that could be distinguished by visual inspection were separated by the YOLO-seg model, whereas TUM-ParticleTyper 2 tended to represent them as a single region (Fig. 2c and 4; see also all glass-substrate examples in Fig. S4). This suggests that, for the present dataset, the YOLO-seg model provided more effective de-agglomeration than the mechanism implemented in TUM-ParticleTyper 2.

Nevertheless, the YOLO-seg model's de-agglomeration is not without issues. First, the success rate of separation is not 100%. Second, even when separation is successful, unusual segmentation boundaries can sometimes be observed (Fig. 2c), which may introduce bias into particle characterization. Therefore, from a practical standpoint, particle aggregation should be avoided, *i.e.*, the dispersion of particles on the substrate should be maximized. Effective strategies to achieve this include down-sampling to reduce the number of particles in suspension prior to deposition on the substrate.

**Fig. 5:**
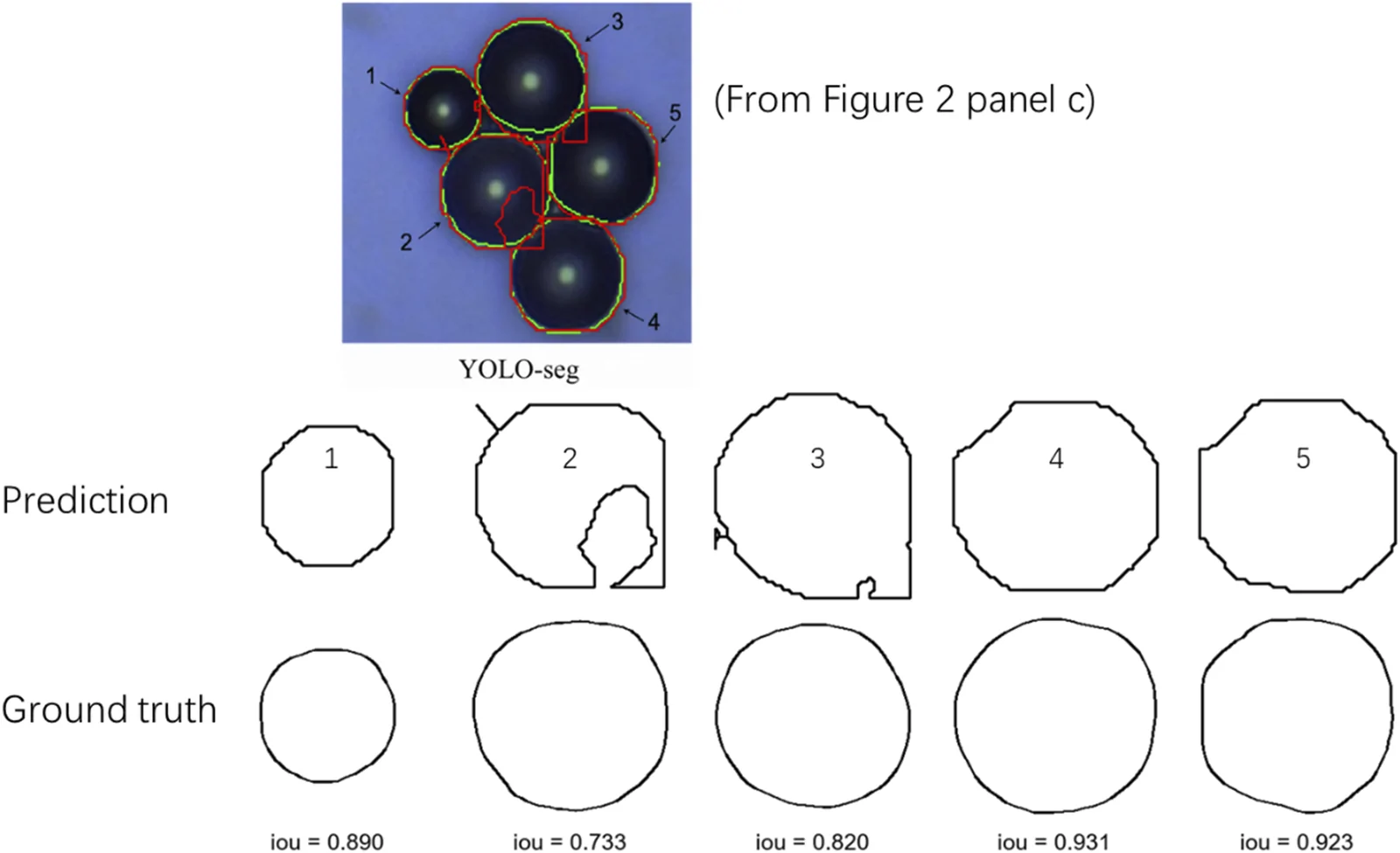
Local contour comparison of YOLO11-predicted and ground-truth boundaries for the five tightly contacting particles in Fig. 2c.

To better examine the unusual segmentation boundaries shown in Fig. 2c, we separately extracted the five tightly contacting, nearly circular particles in this panel and compared the YOLO-seg model predicted contours with the corresponding ground-truth contours (Fig. 5). The comparison showed that the contour deviations were mainly concentrated in particles 2 and 3, whereas particles 1, 4, and 5 showed good agreement between the predicted and ground-truth contours. Particles 2 and 3 still had IoU values of 0.733 and 0.820, respectively, both exceeding the TP criterion used in this study (IoU = 0.70).

Further investigation suggested that this issue was mainly related to the limited effective representation of such nearly circular, tightly contacting particle configurations in the original training set. Although the training set did contain examples of nearly circular particles, images containing three or more tightly contacting nearly circular particles accounted for only 2.1% of the training images (9/437), and the corresponding particles accounted for only 0.9% of all annotated training particles (45/4985). Consistent with this interpretation, when the YOLO-seg model was further fine-tuned using replicated and augmented training images containing similar nearly circular particles, including both isolated particles and tightly contacting multi-particle configurations, the IoU values of the two particles with the most obvious contour deviations in Fig. 2c increased from 0.73 and 0.82 to 0.86 and 0.87, respectively. These results indicate that this local instance-boundary assignment error can be mitigated by increasing the effective training weight of such specific tightly contacting particle configurations. In practical applications, this also suggests that when a particular particle morphology or aggregation pattern is underrepresented in the training data, targeted data collection or augmentation can be used to improve contour precision for that case.

In addition to the comparison with a threshold-based automated workflow, we further compared YOLO-seg with a deep-learning-based instance segmentation method. Mask R-CNN was selected as the benchmark because it is one of the most commonly used methods in MP identification and segmentation studies and has shown good performance in these tasks.^14,18–21^

A Mask R-CNN model was trained and evaluated under the same data split as the YOLO-seg model. Specifically, the same training set and validation set were used for model development, and the final performance was evaluated on the same test set. A summary of the Mask R-CNN training process is shown in Fig. S6. Overall, both the training loss and validation loss decreased rapidly at the early stage and then gradually entered a stable region. The validation mask AP and box AP increased rapidly within the first 20–40 epochs, followed by slower improvement and a late plateau. The best checkpoint was obtained at epoch 89, indicating that the model had reached a converged state.

The trained Mask R-CNN model performed well on the test set, and its predicted contours were generally consistent with the ground-truth contours (Fig. S7). Quantitatively, at IoU = 0.7, Mask R-CNN achieved a precision, recall, and F1-score of 0.85, 0.84, and 0.84, respectively. Its precision was very close to that of YOLO-seg (0.86), indicating that the two models had a similar overall strong ability to suppress obvious background interference. In contrast, the difference in recall was more pronounced: YOLO-seg achieved a recall of 0.93, whereas Mask R-CNN achieved 0.84. Accordingly, the F1-score of YOLO-seg was also higher than that of Mask R-CNN (0.89 *vs.* 0.84). These results indicate that YOLO-seg detected more true particles while maintaining a similar precision. More specifically, 76 particles were successfully detected by YOLO-seg but did not pass the IoU = 0.7 criterion in Mask R-CNN.

To further examine this difference, we analyzed these 76 particles. The results showed that the additional misses of Mask R-CNN were mainly concentrated in three observable error modes. The first was a complete miss, in which a true particle was present in the image but the model produced no final prediction region overlapping that particle; this was the most common error mode and occurred in 31 cases. The second was insufficient contour coverage, in which the model did generate a prediction for the target particle, but the overlap between the predicted contour and the ground-truth contour was not sufficient to meet the IoU = 0.7 criterion; this occurred in 23 cases. The third was merged segmentation of adjacent particles, in which several closely neighboring particles were grouped into a single predicted instance, preventing an individual particle from achieving sufficient overlap with the ground truth; this occurred in 16 cases. In addition, a small number of cases showed fragmented predictions, in which a single particle was split into multiple small predicted regions and none of them individually met the IoU threshold.

Overall, the trained YOLO-seg model performed better than both the conventional threshold-based method and the Mask R-CNN model. The improvement was more pronounced compared with the threshold-based method, while the advantage over Mask R-CNN was smaller but still evident.

#### 3.1.2 Application to real-world environmental samples

By passing tap water through a silicone membrane, an environmental sample was prepared. Then, 1359 optical micrographs were acquired from different regions of the membrane.

Fig. S8(a) shows one of the images collected from the tap water sample. In Fig. S8(a), five visible particles, indicated by green arrows, can be observed. The filter's square pores (each sized 5 µm × 5 µm) are also clearly visible, along with several elongated scratch-like features, which may be fibers, although this cannot be confirmed from the image alone.

Upon noticing the large pores, we hypothesized that applying the trained YOLO-seg model to this image, or to the full environmental image set, would not only detect true particles but also misclassify pores as particles. Inference results (Fig. S8(b)) confirmed this: many FPs occurred as all the 5 µm × 5 µm pores were identified as particles, which is not acceptable.

To address this, we used transfer learning (a technique that is commonly used in deep learning, especially in CV tasks). This approach can keep training costs low while improving generalization, because it adapts the model to new backgrounds and appearances with limited new data. From the 1359 images acquired from the tap water sample, we randomly selected about 10% as a small subset for transfer learning. Within this subset, 96 images (70%) were used as the transfer learning training set and 40 images (30%) were used as the validation set. We also cropped regions that contained only background and no visible particles from images in this 10% subset, and added 13 such crops to the training set and 6 crops to the validation set, in order to strengthen the model's ability to recognize and suppress this new background pattern.

During transfer learning, we froze the backbone (parameter: freeze = 10) so that its feature extractor remained stable. Freezing the backbone preserves the general edge and texture features learned during the original training, reduces the risk of forgetting what the model had already learned, and reduces the number of parameters that need to be updated, which helps to control the training cost.^29,30^ We then fine-tuned the remaining layers on the new images with a very low learning rate. After the losses had converged, we obtained a fine-tuned model that matched the base model on the original test set: mAP@0.5 remained 0.96, and mAP@0.5 : 0.95 decreased only slightly by 0.01. These results indicate that transfer learning successfully improved the model's adaptation to the new background while almost completely avoiding a loss in overall detection performance on the original task. Note that we also found that enabling rotation augmentation during the transfer learning was essential. We set the rotation parameter degrees to 180.0, which applies random rotations in the range from −180° to 180° during training. This setting breaks the bias introduced by a single dominant orientation of the square pores in the fine-tuning images. Without this augmentation, the fine-tuning set is small, and the pore orientation is almost fixed, so the model tends to overfit to that orientation. When the orientation of the square pores in the inference images is different from that in the fine-tuning set, the model again tends to misclassify background pores as particles. With degrees = 180 enabled, the model becomes robust to pore orientation (approximately rotation invariant), and these orientation-driven misclassifications disappear.

We then applied the fine-tuned model to the remaining 90% of the tap water images, that is, 1223 micrographs. For each image, we overlaid the contours of the predicted segmentation masks and the manually annotated ground truth masks on the original optical image for visual inspection, as illustrated in Fig. S9. Using an IoU threshold of 0.7, the evaluation gave 2535 TPs, 735 FPs and 290 FNs, corresponding to a recall of 0.90, a precision of 0.78 and an F1 score of 0.83. To evaluate how well the model could at least locate particles without requiring accurate overlap, we repeated the analysis with a very low IoU threshold of 0.01. In this case, the counts became 2664 TPs and 161 FNs, and recall increased to 0.94. Taken together, these results indicate that the model was able to find the vast majority of particles, but some predicted masks did not meet the stricter requirement of IoU = 0.7 with the ground truth.

The 735 FPs are nevertheless a relatively large number, so we examined them in more detail. The inspection showed that they could be grouped into three categories. The first category contained 91 cases of redundant splitting of particles. In these cases, more than one predicted mask overlapped the same ground truth particle with an IoU of at least 0.7. In almost all of these 91 cases, one relatively large prediction traced the particle well, while a smaller prediction lay almost entirely inside this larger region, as revealed by visual inspection. Under the evaluation rule, the larger mask was counted as the correct detection, while the smaller nested mask was treated as an extra detection and therefore contributed to the FP count. The second category comprised 258 masks with no overlap at all with any ground truth mask. The remaining 386 FPs arose from predictions that did overlap a real particle but had an IoU below 0.7. Within this group, a noticeable subset again showed a large–small pattern in which a larger mask already covered most of a particle and an additional smaller fragment appeared inside or around its boundary. These small fragments did not reach an IoU of 0.7 with the corresponding ground truth mask and were therefore counted as IoU-insufficient FPs.

A part of these FPs can be removed by using simple prior knowledge from MP practice and applying a postprocessing step to the segmentation output. In routine sample preparation, MP particles deposited on the substrate are deliberately spread out as much as possible so that they do not sit very close to each other or form obvious stacks. In practical terms, once a compact segmented region that looks like a particle is present in an image, it is usually reasonable to interpret this region as a single MP particle, without further considering the possibility that it is a large particle with one or several intact smaller particles stacked on top of it. On this basis, we can apply a simple rule to the model output. Wherever a local group of masks shows a clear pattern where a larger mask almost completely contains a smaller one, this group can be treated as alternative segmentations of the same particle. The larger mask is kept, and the smaller, contained mask is regarded as a redundant fragment and removed. This physics-motivated and experimentally motivated postprocessing step removed 346 of the 735 FPs, which corresponds to 47% of all FPs. As a result, precision increased from 0.78 to 0.87 and the F1-score increased from 0.83 to 0.88. After this postprocessing, we overlaid the contours of the resulting segmentation masks together with the contours of the ground truth masks on the original optical images for visual inspection (Fig. S10).

**Fig. 6:**
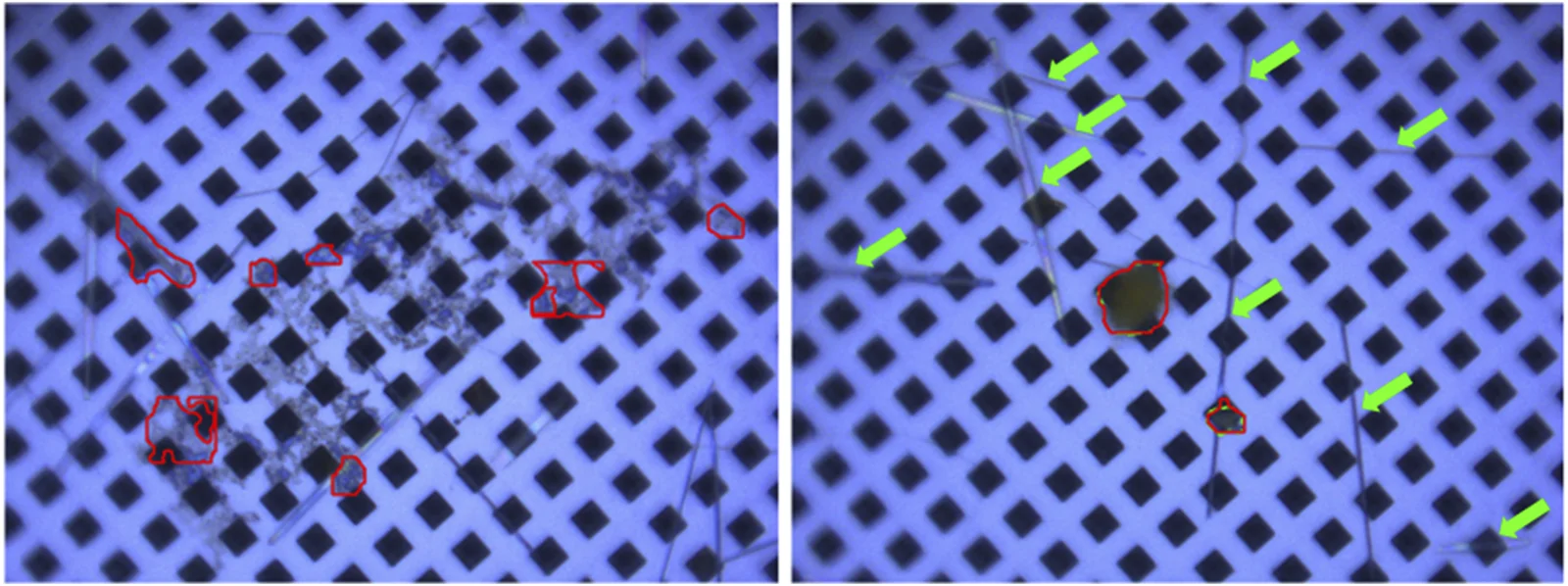
Optical micrographs of the silicone membrane used to filter tap water in this study. Two example micrographs are provided. Green: ground truth contours; red: segmentation contours generated by the YOLO-seg model. The arrows in the image on the right point to the fibrous structures in that image.

Among the 258 FPs that occurred in regions without any ground truth annotation, most arose because the model interpreted stains like traces on the filter surface, such as water marks or residual contamination (Fig. 6, left), as particles.

Unlike experiments using reference particles/fibers, where the materials are dispersed over a clean and uniform background, environmental samples often present backgrounds containing stain-like traces, as shown in Fig. 6. Indeed, in real-world environmental samples, any material may be present, including substances that interfere with the analysis. The features shown in Fig. 6 may include inorganic residues left after drying (for example, salt) as well as trace amounts of organic matter. Appropriate sample pretreatment that removes as much non-MP material as possible would plausibly reduce such interferences and thereby lower the FP rate.

From the model's perspective, however, the FPs observed in Fig. 6 are largely attributable to the domain shift between training and deployment. Again, our detector was trained mostly under idealized conditions, using reference MPs dispersed on a clean filter background. In contrast, real environmental samples exhibit stain-like traces and heterogeneous residues that were underrepresented in the training data. Because the detector learns appearance-based decision boundaries from the training distribution, these previously unseen background textures, which may share visual cues with MPs (*e.g.*, edges, contrast, elongated shapes), can be assigned high objectness scores and thus be misclassified as particles in real-world images. Importantly, this is a data-driven rather than a fundamental limitation. Incorporating representative environmental images, particularly those containing typical stain-like backgrounds, into continued training (fine-tuning) or re-training would provide hard negative examples and progressively calibrate the model to recognize such traces as background, thereby reducing the FP rate in practical applications. Consistent with this view, prior deep-learning-based CV studies for MP detection under microscopy images have shown that including complex and realistic backgrounds in the training set improves robustness at test time and mitigates background-induced FPs.^21,31^

In addition to particles, it could be observed that the silicone membrane used in this study also contains fibrous structures (Fig. 6, right; green arrows point at the observed fibrous structures). Some appear to be true fibers, whereas others may be scratches produced during filtration by hard objects in the water. In this study, the YOLO-seg model was trained on images of particles and fragments with modest aspect ratios, and these images contained almost no fibers. As a result, the learned morphological priors are aligned with particle- and fragment-like shapes, and the model could not detect fibers. Accordingly, we did not annotate fibrous structures in the micrographs of the tap water sample, and the model also ignored most of them. Only a very small fraction of these fibrous structures triggered detections, where portions of a fiber were interpreted as particles or fragments.

**Fig. 7:**
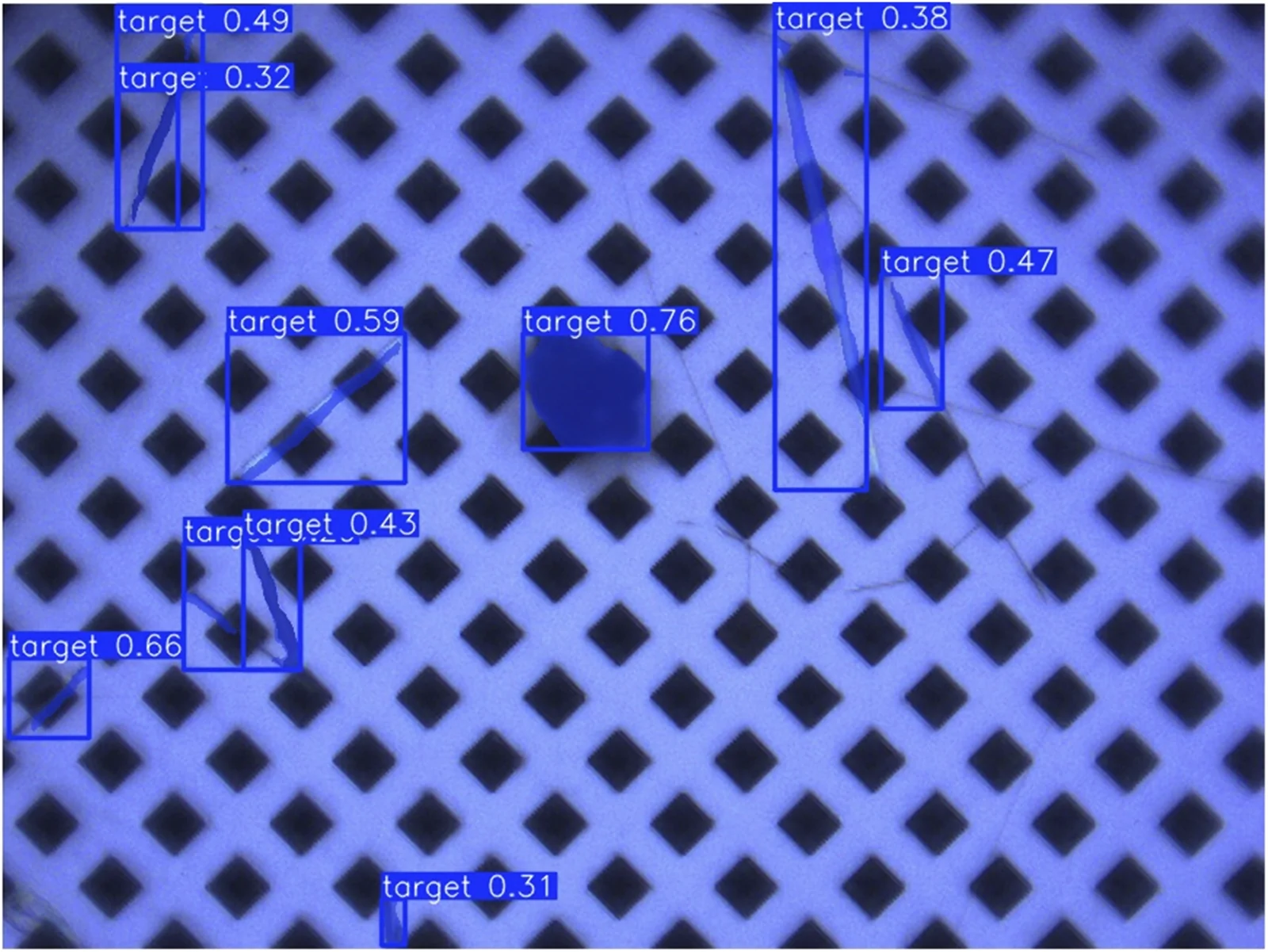
YOLO-seg detections on a micrograph containing both particles and fibers.

If the initial training had included images containing fibers, the model could have learned to detect them.^32,33^ At the time, suitable fibrous reference materials were not available. We expected this gap to be addressed through transfer learning. After performing transfer learning with a small number of micrographs from the tap water sample that contained fibrous structures, the model learned to recognize fibers (Fig. 7). This experiment served as a minimal proof of feasibility.

**Fig. 8:**
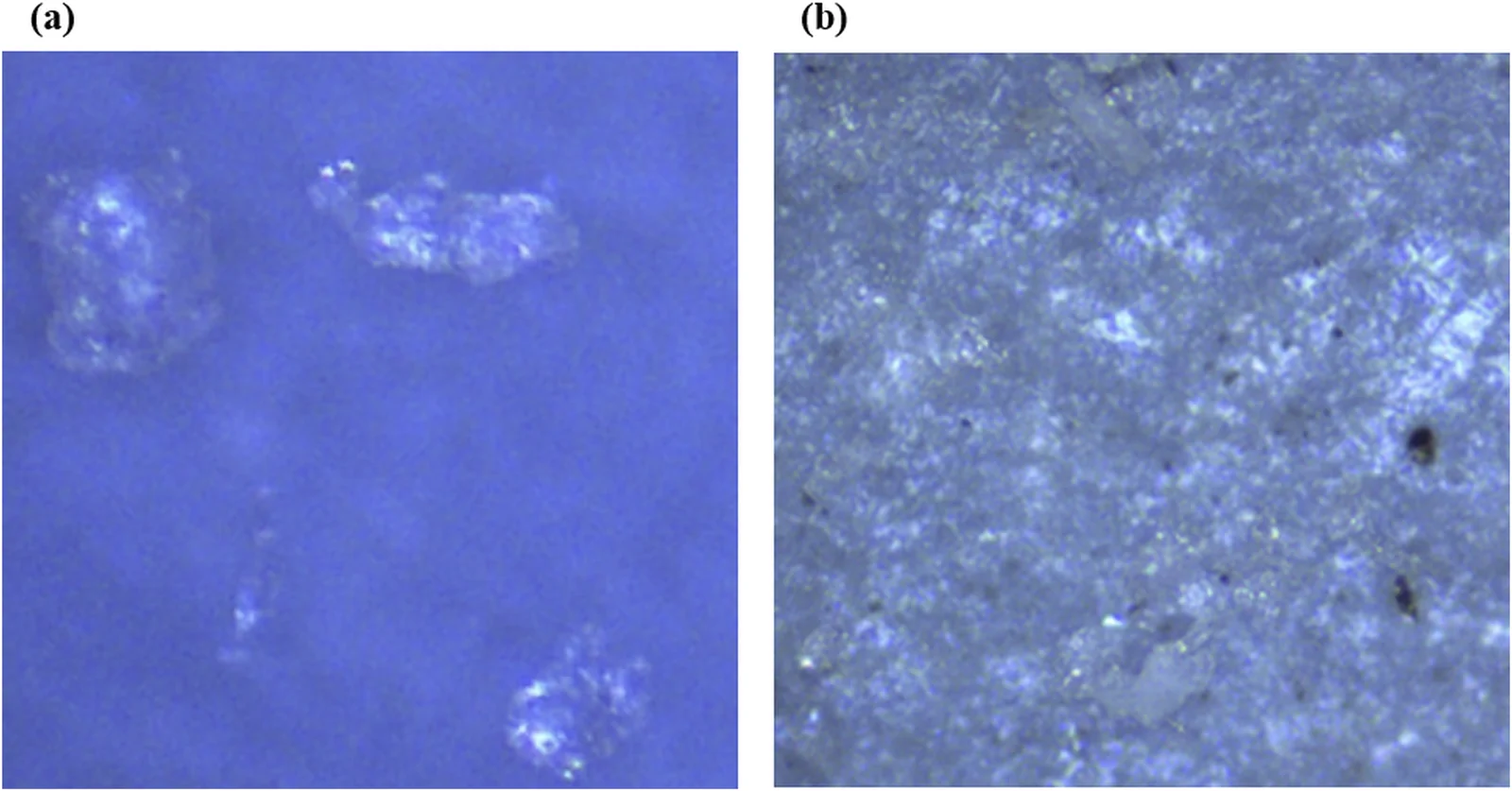
Difference in the Al_2_O_3_ filter background between a reference particle sample and a commercial salt sample. (a) Reference microplastic particles deposited on an Al_2_O_3_ filter. (b) Al_2_O_3_ filter after filtration of the processed commercial salt sample.

In addition, a sample derived from commercial salt was included. After preprocessing, this sample was deposited on an Al_2_O_3_ filter. Compared with the reference particle sample or the tap water sample, filtration of the salt sample produced a background with much more ‘interference’, making particle boundaries more difficult to distinguish in the optical images. Fig. 8 shows this difference in background appearance. For the Al_2_O_3_ filter sample prepared with reference MPs, the filter background was clean and uniform, and the four reference particles on the filter were easily identifiable (Fig. 8a). By contrast, the sample obtained from the commercial salt showed an obviously dirty background, and the boundaries between particles and the surrounding filter background were difficult to distinguish (Fig. 8b).

The Al_2_O_3_ filter images obtained from the commercial salt sample showed background features that differed clearly from those in the original training images. As described above, this background difference introduces a distribution shift and affects the direct application of the original model to real sample images. To improve the model's adaptation to images with this dirty filter background, five microscopy images from the commercial salt sample were used for a lightweight transfer learning step. The adapted model was then applied to test images acquired from the same filter that were not used during model fine tuning, and its quantitative performance is summarized in Table 5.

**Table 5:** Segmentation performance of the fine-tuned YOLO-seg model on test images from the commercial salt sample. IoU, intersection over union; TP, true positives; FN, false negatives; FP, false positives; P, precision; R, recall; F1, F1 score

IoU threshold	TP	FN	FP	P	R	F1
0.7	90	69	61	0.60	0.57	0.58
0.6	106	53	45	0.70	0.67	0.68
0.5	115	44	36	0.76	0.72	0.74

As shown in Table 5, under the stricter IoU threshold of 0.7, the model produced 90 TPs, 69 FNs, and 61 FPs, with P, R, and F1 values of 0.60, 0.57, and 0.58, respectively. When the IoU threshold was relaxed, model performance improved. At an IoU threshold of 0.6, the number of TPs increased to 106, while FNs and FPs decreased to 53 and 45, respectively, with P, R, and F1 values of 0.70, 0.67, and 0.68. At an IoU threshold of 0.5, the number of TPs further increased to 115, while FNs and FPs decreased to 44 and 36, respectively, with P, R, and F1 values of 0.76, 0.72, and 0.74. This indicates that the model was still able to identify a substantial proportion of particles in the complex Al_2_O_3_ filter background produced by the salt sample. At the same time, the results were sensitive to the IoU threshold, suggesting that particle boundaries in this type of complex filter background were more likely to become visually ambiguous, leading to local shifts or insufficient overlap between predicted contours and manual annotations.

**Fig. 9:**
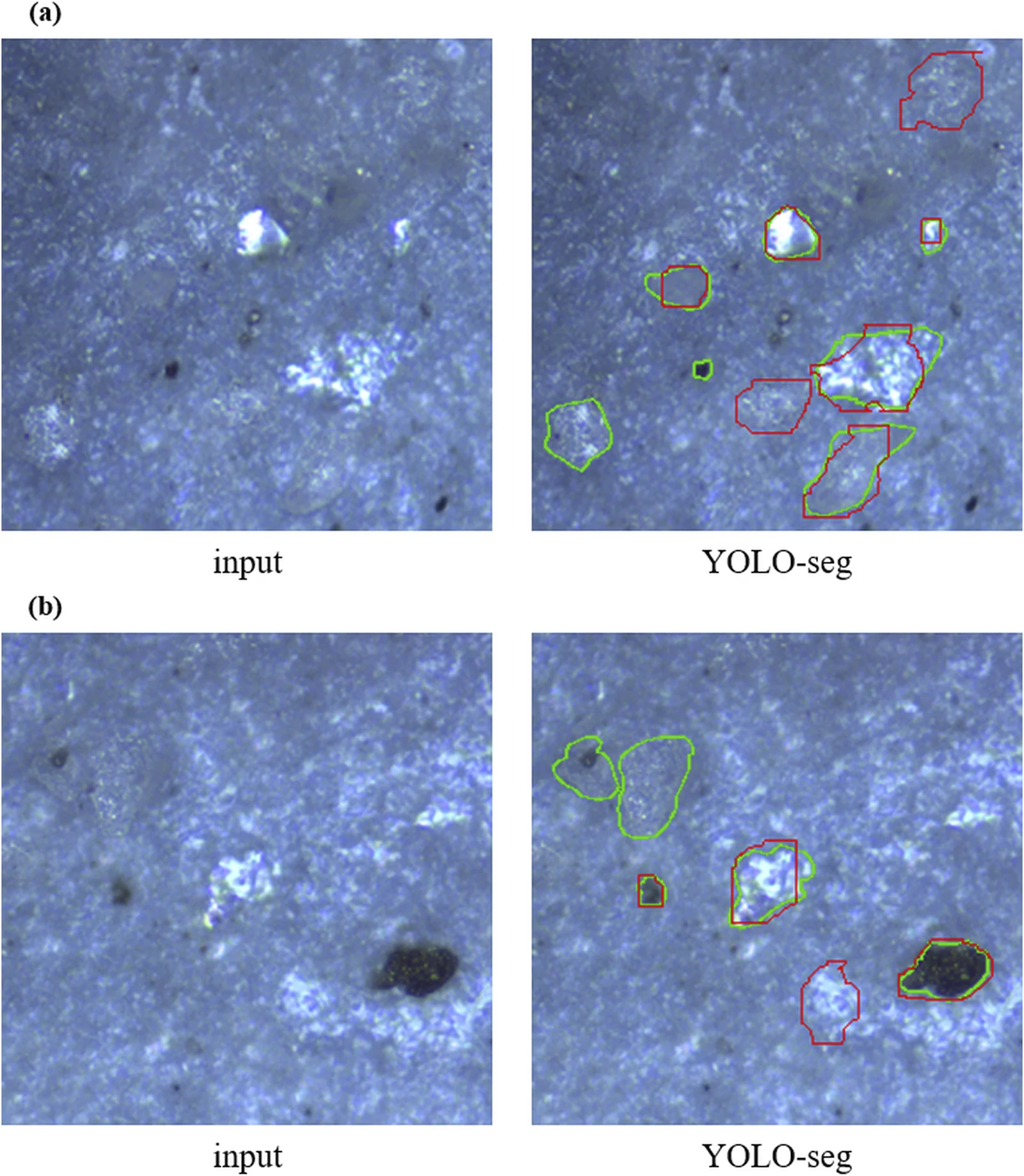
Segmentation contours produced by the adapted YOLO segmentation model on two cropped test regions from the commercial salt sample deposited on an Al_2_O_3_ filter: (a) test region 1 and (b) test region 2. In both (a) and (b), the left panel shows the original optical image, and the right panel shows the same region with overlaid contours. Green: ground truth contours; red: segmentation contours generated by the adapted YOLO segmentation model. Only two cropped regions are shown here; visualized results across all test images from the commercial salt sample are provided in Fig. S11.

The visualized results were consistent with the quantitative results shown in Table 5. In the dirty Al_2_O_3_ filter background, the model generally produced predicted contours near manually annotated particles, but the red predicted contours did not always overlap closely with the green manual annotation contours, indicating that the complex background mainly affected the precise segmentation of particle boundaries. This interpretation was further supported by particles with more distinct visual features: for example, the brightest particle near the center of Fig. 9a and the black particle with strong color contrast against the surrounding background in the lower right region of Fig. 9b both showed close agreement between the predicted contours and the manual annotations. One green contour in the lower left region of Fig. 9a and two similar green contours in the upper left region of Fig. 9b had no corresponding red predicted contours, suggesting that missed detections occurred in the dirty background.

A small number of regions were marked only by red predicted contours. In Fig. 9a, the two red predicted contours without overlap with any green manual annotation contour, one near the center and the other near the upper right region of the overlaid panel, showed an appearance resembling particles in the original image and may therefore reflect differences between manual annotation and model interpretation. This is consistent with the practical difficulty encountered during manual annotation, as some local structures in dirty filter backgrounds were not always easy to classify as particles or background. By contrast, the red predicted contour slightly to the left of the black particle in Fig. 9b appeared more consistent with background residue or texture.

Therefore, the more complex and dirtier filter background posed a clear challenge to the model. When the IoU threshold was relaxed, the model showed better performance in locating particles, but this improvement also meant that the requirement for particle shape and contour agreement was less strict. For images with this type of dirty background, the segmentation results therefore require careful interpretation.

### 3.2 Model output

Applying the YOLO-seg model to an image produces a txt file in which each detected object occupies one line; if no object is detected, the file is empty. Each line begins with the integer class label, followed by an ordered list of polygon vertices (*x*_1_, *y*_1_), (*x*_2_, *y*_2_), …, (*x*_*n*_, *y*_*n*_). All coordinates are normalized to the interval [0,1] with respect to the image width (for *x*) and height (for *y*), meaning that these points are expressed in the YOLO coordinate system. The YOLO coordinate system is defined such that the origin (0,0) is at the top-left corner of the image, the *x*-axis increases to the right up to 1, and the *y*-axis increases downward up to 1.

**Fig. 10:**
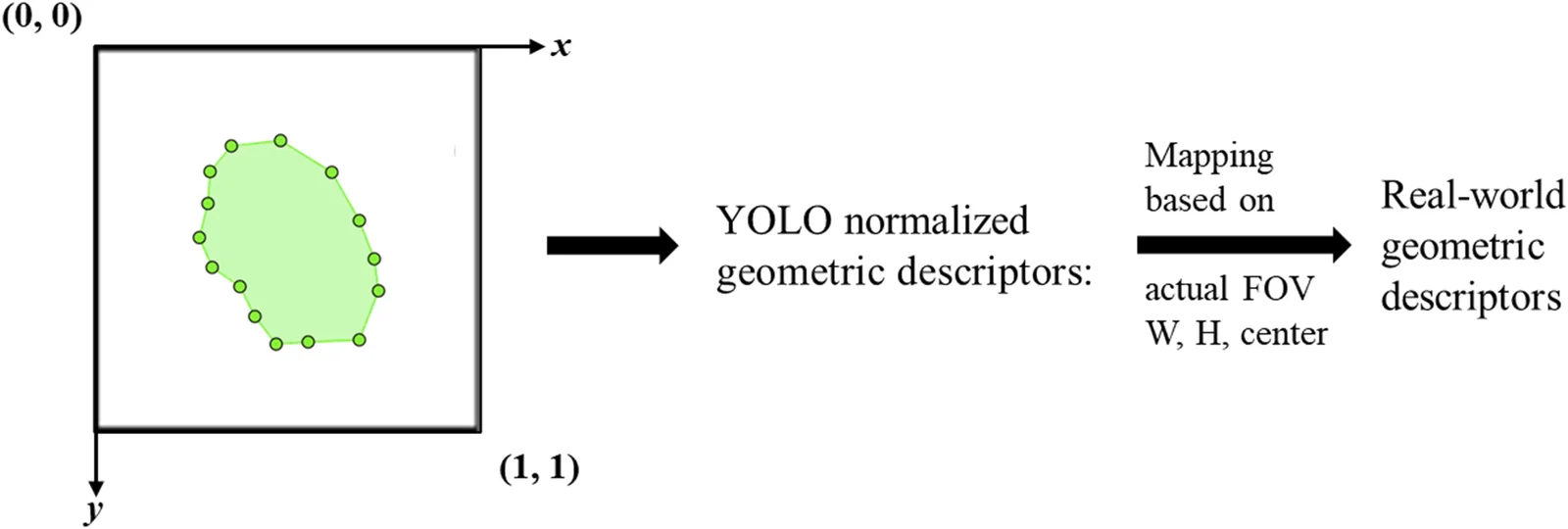
Workflow from the YOLO-normalized coordinate system to real-world geometric descriptors. Mapping quantities computed in the YOLO-normalized coordinate system to real-world geometric descriptors requires the physical field-of-view dimensions (width W and height H). Converting the center of the maximum inscribed circle to microscope sample-stage coordinates additionally requires the true stage coordinates of the FOV center.

As shown in Fig. 10, the YOLO coordinate system is illustrated together with an example prediction consisting of 15 vertices (green points), where the enclosed light-green region represents the segmentation polygon generated by the model. Based on the vertex list (*x*_1_, *y*_1_), (*x*_2_, *y*_2_), …, (*x*_*n*_, *y*_*n*_), a variety of geometric descriptors can be directly computed in the YOLO coordinate system, such as the segmented area, the maximum Feret diameter, the minimum Feret diameter, *etc.* These quantities, initially obtained in normalized coordinates, can then be mapped to real-world measurements through simple transformations using the physical width and height of the field of view (Fig. 10), thereby enabling quantitative characterization of the detected objects.

In MP detection, morphological classification of segmented areas is not only a popular topic but also of considerable importance. For example, morphological traits provide valuable clues for source tracing: fragments are often indicative of the breakage of plastic products, whereas fibers are more likely to originate from textiles. Likewise, different morphologies correspond to distinct environmental behaviors and can be used to predict patterns of transport, settling, and dispersion in aquatic systems or sediments. Moreover, morphology influences biological uptake and toxicological effects.

Previous studies distinguish fibers from particles using morphological features computed from segmented areas, such as max/min Feret diameters, aspect ratio, circularity, convexity, and so on. Some studies build simple models on subsets of these features for shape classification,^6,8,34^ while others adopt more sophisticated approaches. For example, Jacob *et al.*^11^ used a random forest classifier trained on six features extracted from segmented areas to classify “fiber *vs.* particle”, reporting performance comparable to human judgment.

With the advancement of deep learning-based CV, a number of studies have recommended adopting an end-to-end CV approach: using expert-provided, high-quality annotations as supervision and allowing neural networks to automatically learn multi-scale geometric and contextual cues directly from raw images and segmentation masks.^13,20,27,28,35^ This strategy avoids predefined, hand-selected features, thereby simplifying the workflow. Moreover, with sufficient capacity and proper training, deep networks can approximate complex nonlinear decision boundaries, enabling robust handling of C/S/U-shaped curved fibers and self-entangled cases, for example as demonstrated in the study of Lorenzo-Navarro *et al.*^28^

Our current model, as a deep learning CV model, can be extended to such morphological classification; however, we do not output explicit “fiber/particle” labels, primarily because at an early stage of model development, we did not have suitable fibrous reference materials.

The YOLO-seg model developed in this study is designed not only for particle detection and characterization but also for determining a representative sampling point within each segmented object for subsequent automated spectral data acquisition. To this end, we propose selecting the center of the maximum inscribed circle of the segmentation area as the sampling point. This approach ensures that the chosen point is strictly located inside the segmentation region and effectively avoids boundary artifacts. Importantly, it is applicable not only to regular particles but also to irregular morphologies such as fibers, ring-shaped (“donut-like”) structures, and dumbbell-shaped objects. Geometrically, this point provides a representative spatial position of the detected object.

The center of the maximum inscribed circle (*x*_c_, *y*_c_) is defined as the point within the segmentation polygon 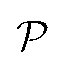 that maximizes the minimum distance to all polygon edges:
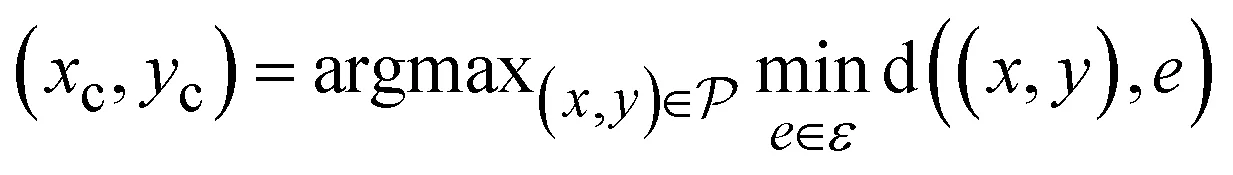
where *ε* denotes the set of polygon edges, and d ((*x*, *y*), *e*) is the Euclidean distance from point (*x*, *y*) to edge *e*.

Once this point is computed in the YOLO coordinate system, it can be mapped to real stage coordinates using the known physical dimensions of the field of view and the true stage coordinates of the field-of-view center (Fig. 10).

### 3.3 Time efficiency of the automated workflow

The time efficiency of the automated workflow is mainly affected by several practical factors, including the area of the ROI, the magnification/field of view of the objective, the hardness and flatness of the substrate, and the inference speed of the YOLO segmentation model, which depends on computational power.

(1) Area of the ROI. In our automated workflow, the microscope scans the sample surface step by step using a motorized stage, and each optical frame covers only a single microscopic field of view. To image a macroscopic ROI, many adjacent fields must be recorded and then stitched together in a mosaic-like manner, as illustrated in Fig. S1. As a result, the image-acquisition time is directly proportional to the ROI area and to the number of fields required to cover that area.

(2) Magnification and field of view. The IR microscope used in this work is equipped with two objectives whose physical fields of view are approximately 639 µm × 480 µm and 166 µm × 124 µm. The first objective has lower magnification and a larger field of view. The second objective has higher magnification and a smaller field of view and can provide higher spatial resolution, but the number of fields that must be recorded to cover a given ROI increases substantially, and hence the data collection time becomes much longer.

(3) Hardness and flatness of the substrate. The hardness and flatness of the substrate directly determine whether per-field refocusing is required. When MPs are concentrated on a hard and flat substrate, the sample height remains nearly constant as the motorized stage moves, so in most cases the focus does not need to be adjusted for every field. On an uneven substrate, however, small height variations can cause defocus (even when a filter holder is used). In such cases, several focal planes are recorded for each field, and YOLO-based inference is used to select the sharpest image for subsequent analysis. This z-stack acquisition increases the total time approximately in proportion to the number of focal planes recorded per field.

(4) Inference speed of the YOLO segmentation model. Once the optical images have been acquired, the inference speed of the YOLO segmentation model determines how long it takes to complete automatic particle recognition. Inference speed refers to the time required for a trained YOLO model to perform one forward pass on a single image. This quantity depends mainly on whether a graphics processing unit is used and on the computing power and memory capacity of that unit.

As a practical example, we processed an approximately 144 mm^2^ ROI on an aluminum oxide filter using the 10× objective (based on an effective membrane diameter of approximately 21 mm for this filter, this ROI accounted for about 42% of the useable membrane area). The total time from optical image acquisition to YOLO-based detection and target-position generation (*i.e.*, generation of the (*x*, *y*, *z*) coordinates for subsequent spectral acquisition of each particle) was approximately 3.1 h. Under the 10× objective, each microscope camera image covered an area of 639 µm × 480 µm. Therefore, 470 camera fields were required to cover the 144 mm^2^ ROI. Because the aluminum oxide filter was not completely flat under microscopic observation, local defocus could occur after the stage moved to different positions in the *x-y* plane. Hence, for each camera field, 11 z-stack images were acquired at a step size of 2 µm to allow subsequent selection of the clearest focal plane. The z-stack acquisition time for each camera field was approximately 18 s; this time included the short waiting period after each z-position adjustment to ensure a stable response from the microscope-control software and motorized stage. Thus, z-stack image acquisition for the whole ROI required about 141 min. Stage movement between adjacent camera fields added approximately 24 min, including both the stage movement itself and the waiting time used to ensure a stable response from the microscope-control software and motorized stage. In addition, YOLO inference for the resulting 5170 images required approximately 22 min on an NVIDIA T1000 GPU with 4 GB VRAM (253 ms per image). The subsequent focal-plane and target-position selection step, which selected one optimal *z*-plane from the 11 z-stack images for each camera field, required only about 3.4 s. Therefore, the total time was approximately 187 min, or about 3.1 h.

It should be noted that YOLO itself is a real-time detection technique, but the implementation described above did not fully exploit this real-time capability. Owing to the current computational resources, this study adopted a processing mode in which z-stack images were first acquired and YOLO inference and target-position generation were then performed on the saved images. With higher computing power, YOLO could be run in real time on a continuous image stream during *x-y* stage movement and *z*-direction focusing movement, allowing particle detection, optimal focal-plane selection, and three-dimensional coordinate extraction to be performed synchronously during image acquisition, which would greatly improve efficiency.

After the (*x*, *y*, *z*) target positions are generated, these coordinates are passed to the microscope software for subsequent spectral acquisition of each particle. The time required for this subsequent step depends on the number of particles detected in the ROI. Under the settings used in this study, the instrument requires approximately 15 s to complete one single-point measurement; the specific parameters were spectral range = 1801–769 cm^−1^, number of scans = 3, and spectral resolution = 2 cm^−1^. These parameters are major factors influencing the speed of spectral acquisition.

### 3.4 Automated refocusing for individual particles

In this work, once the spectrum acquisition coordinates (*x*, *y*, *z*) of particles in the microscope are confirmed, the next step is to move the motorized stage to enable spectrum collection at these (*x*, *y*, *z*) coordinates.

However, for acquiring spectral data of the highest quality, the optimal approach is to first automatically optimize the focal distance (*z*) for individual particles before spectrum collection. This is because, through YOLO inference, each particle within an area viewed by the microscope camera is assigned a (*z*) value that ensures most particles in that region are visibly in focus; however, this determined (*z*) may not necessarily be the most suitable for obtaining high-quality spectra. A method to refocus for each particle is needed to deal with this situation.

The Auto focus function based on QCLs is a fast and effective method for fine-tuning the focal distance of individual particles. The principle of Auto focus is that, after a particle has been assigned a (*z*) and an Auto focus range is selected (*e.g.*, *z* ± 50 µm), the particle is probed for its response to IR light at a user-specified frequency/wavenumber within the Auto focus range; once this is done, the focal distance at which the particle exhibits the maximum IR response can be identified. This focal distance can then be considered as the optimal focal distance for acquiring spectra. Note that the user-specified frequency used in Auto focus must be a frequency at which the IR light of this frequency can induce an IR response of the particle when in focus. Fig. S12 shows the results of using Auto focus for focal distance optimization of a 15 µm nylon particle. In this Auto focus process, the frequency of the IR light used was 1639 cm^−1^, which is a characteristic frequency of nylon (IR light at 1639 cm^−1^ can induce a strong IR response of nylon when in focus). In the graph, the *x*-axis value of 2780 µm represents the original focal distance of this nylon particle. Although the particle appeared visibly clear at 2780 µm, its response to IR light at 1639 cm^−1^ was basically 0, indicating that 2780 µm was not an optimal focal distance for spectrum collection for this particle. Auto focus for this nylon particle was conducted within an Auto focus range of 2730 µm to 2830 µm (*i.e.*, 2780 ± 50 µm). The data produced represented the IR response of the nylon particle to IR light of 1639 cm^−1^ within this range, as shown in Fig. S12. It can be seen that at a focal distance of 2768 µm (marked by the orange line in Fig. S12), the nylon particle exhibited the highest IR response to IR light at 1639 cm^−1^. Additionally, the signal-to-noise ratio (SNR) in this Auto focus graph is high. Hence, 2768 µm was determined by Auto focus as the optimal focal distance for acquiring spectra for this nylon particle.

In an automated MP detection workflow using the Auto focus function for focal distance optimization, a crucial principle is to choose a suitable frequency or frequencies. An ideal situation is one in which the types of plastic in the sample are known, and the analyst is accordingly able to select a frequency or frequencies that can induce IR responses from the particles in the sample. However, in many cases, the plastic types in the sample are unknown. In such situations, the selection of frequencies should consider all common plastic types.

Certainly, applying the Auto focus function extends the analysis time for each particle by *α* seconds *β, where *α* seconds represents the time needed to perform Auto focus once, and *β* represents the number of times Auto focus is applied to a particle (1 ≤ *β* ≤ the number of selected frequencies). This compromise in time efficiency is justifiable in exchange for obtaining high-quality spectral data. However, it is important to note that the need to refocus for individual particles (or the use of Auto focus) may be instrument-specific. In our investigation, we exclusively examined the mIRage IR microscope and observed its heavy reliance on Auto focus. Jacob *et al.*^11^ reported that the WITec Raman microscope system also provides a “Spectral Auto Focus” function, although it remains unclear whether this system exhibits the same level of dependence on Auto focus as observed in the mIRage IR system.

## 4 Conclusion

With increasing scientific and public concern about MP pollution, there has been an urgent need for routine and large-scale monitoring of MPs, and automation is essential to meet this demand. Here, we present a novel, high performance automated workflow for MP detection centered on the Ultralytics YOLOv11 segmentation model. This workflow has the potential to support larger-scale and more complex MP analysis, thereby facilitating more comprehensive investigations into the distribution, characteristics, and ecological implications of MPs in the environment.

The core of the proposed workflow is a deep-learning-based CV model, and this model should not be regarded as a fixed, once-and-for-all solution. In real-world industrial applications of CV, models are rarely deployed as final and permanently unchanged systems. In fields such as logistics sorting,^36^ quality inspection,^37^ autonomous driving,^38^ facial recognition,^39^ and so on, CV models are continuously updated to learn new knowledge as new data become available, and transfer learning provides an effective approach for updating them,^40^ which is why it has become a cornerstone of modern deep learning.

This is brought up to highlight that, in the task of applying a CV model to microscopy images for identifying those objects that “to the eye” appear worth characterizing and acquiring spectral for chemical identification, the model likewise needs to be continuously improved. While the “base model” we provide here is useful, there remains room for enhancement. In the future, when encountering images of particles/fibers with a broader range of physical properties (*e.g.*, diverse colors, shapes, and sizes) placed on a wider variety of substrates (*e.g.*, silver membranes, gold-coated filters, calcium fluoride windows, and glass fiber filters), applying transfer learning will further improve the model's performance.

To sustain such progress, an image database should be established, where MP researchers can upload their well-annotated image data. This would provide a continuous stream of high-quality samples for updating CV models, in the same way that the established Open Specy spectral database enables ongoing improvements in spectral analysis.^41,42^ Several studies have released high-quality, well-annotated datasets. Notably, Lee *et al.*^43^ published bright-field microscopy images of particles isolated from sewage environments and provided both raw and augmented images for segmentation and detection tasks: 39 953 images for segmentation and 341 723 images for detection. This represents a very substantial volume of data. Looking ahead, consolidating and continually updating an open microscope-image repository that covers both particles and fibers would significantly advance CV research on MPs by improving model generalization and cross-substrate transferability, supporting transfer learning and continual learning paradigms, enabling comparable benchmarking and greater reproducibility, and accelerating model iteration and the translation from laboratory research to real-world applications.

## Conflicts of interest

There are no conflicts to declare.

## Supplementary Material

AY-018-D6AY00142D-s001

## Data Availability

All data and code, and a demo video could be accessed through: https://drive.google.com/drive/folders/1g3lnhXggQYbZrCQPiHk1EXdFZ2zKLQO4?usp=sharing. Supplementary information (SI) is available. See DOI: https://doi.org/10.1039/d6ay00142d.
