# Supplementary material for: Development of a YOLO-guided automated (microplastic) particle analysis workflow
Source: Anal Methods. 2026 Sep 4;18(39):8977–95. doi: 10.1039/d6ay00142d (PMC13543028; doi:10.1039/d6ay00142d)
Supplement: Supplementary file 1 [file AY-018-D6AY00142D-s001.pdf]

This journal is © The Royal Society of Chemistry 2026

Development of a YOLO-Guided Automated (Microplastic) Particle Analysis Workflow

Junhao Xie *, Aoife Gowen, and Jun-Li Xu

School of Biosystems and Food Engineering, University College Dublin, Belfield, Dublin 4, Ireland.
* Correspondence: junhao.xie@ucdconnect.ie, Tel: +353 (0) 87 231 5465

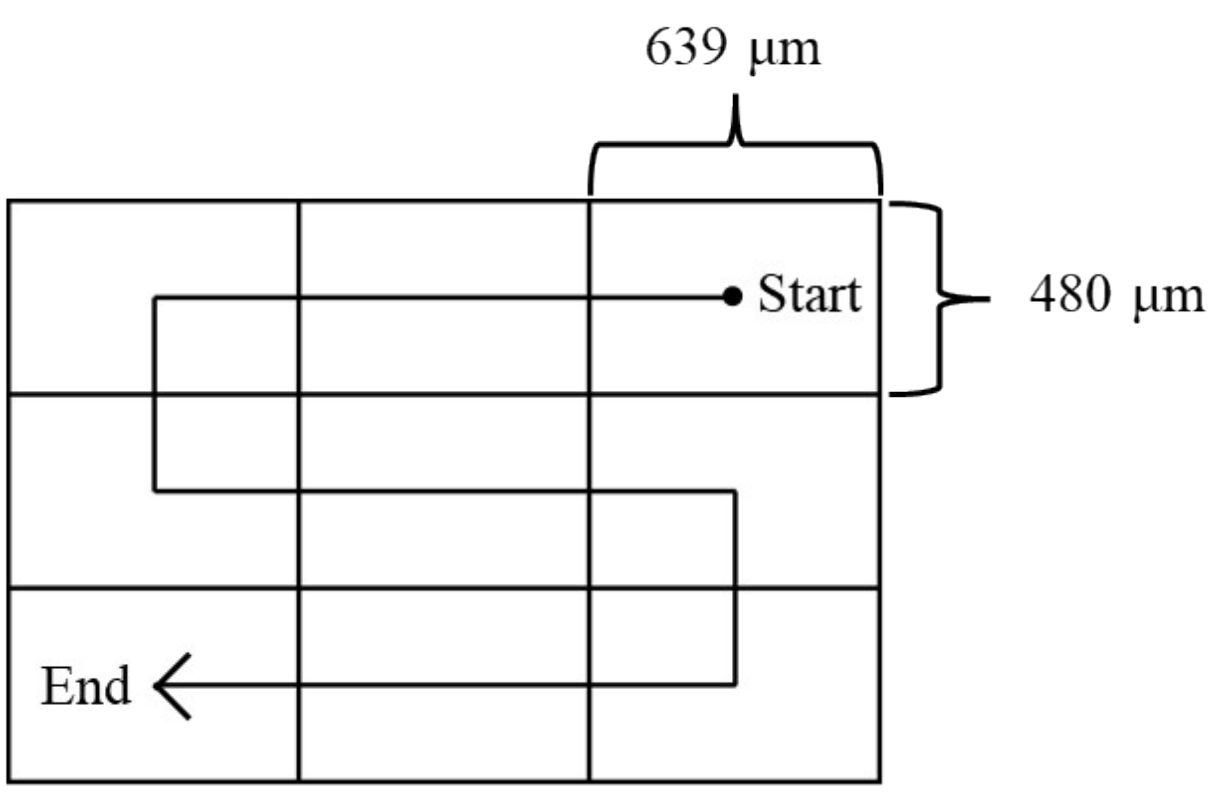

Figure S1. Schematic diagram illustrating how optical images are collected to cover the designated ROI. Image acquisition starts from the top-right corner of the ROI and ends at the bottom-left corner of the ROI. The microscope camera has a field of view of 639 µm (width) × 480 µm (height), so in this illustration the covered ROI corresponds to 9 × (639 µm × 480 µm).

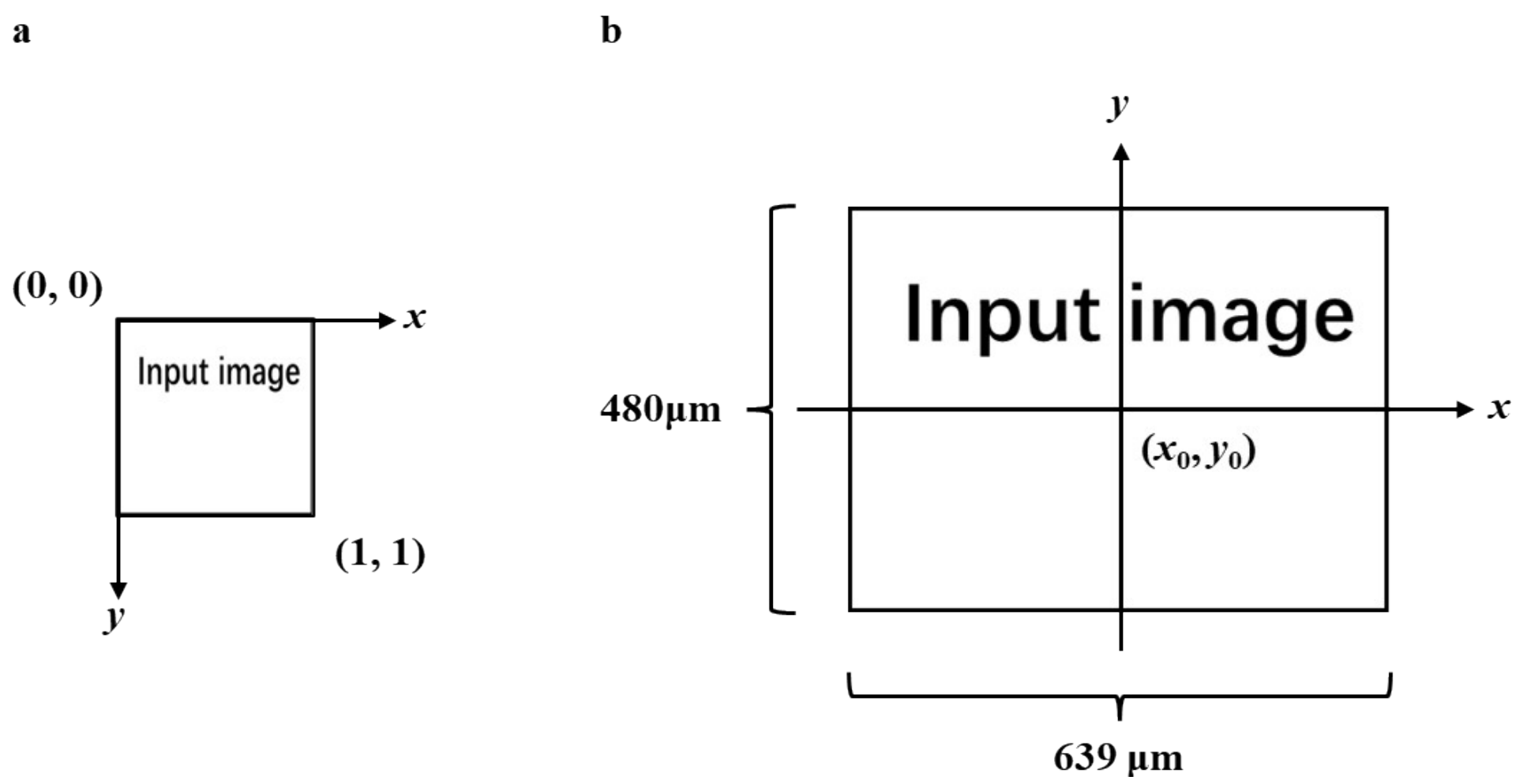

Figure S2. The YOLO coordinate system (a); and a hypothetical microscope single-field coordinate system (639 µm × 480 µm) with field center (x_0, y_0) (b).

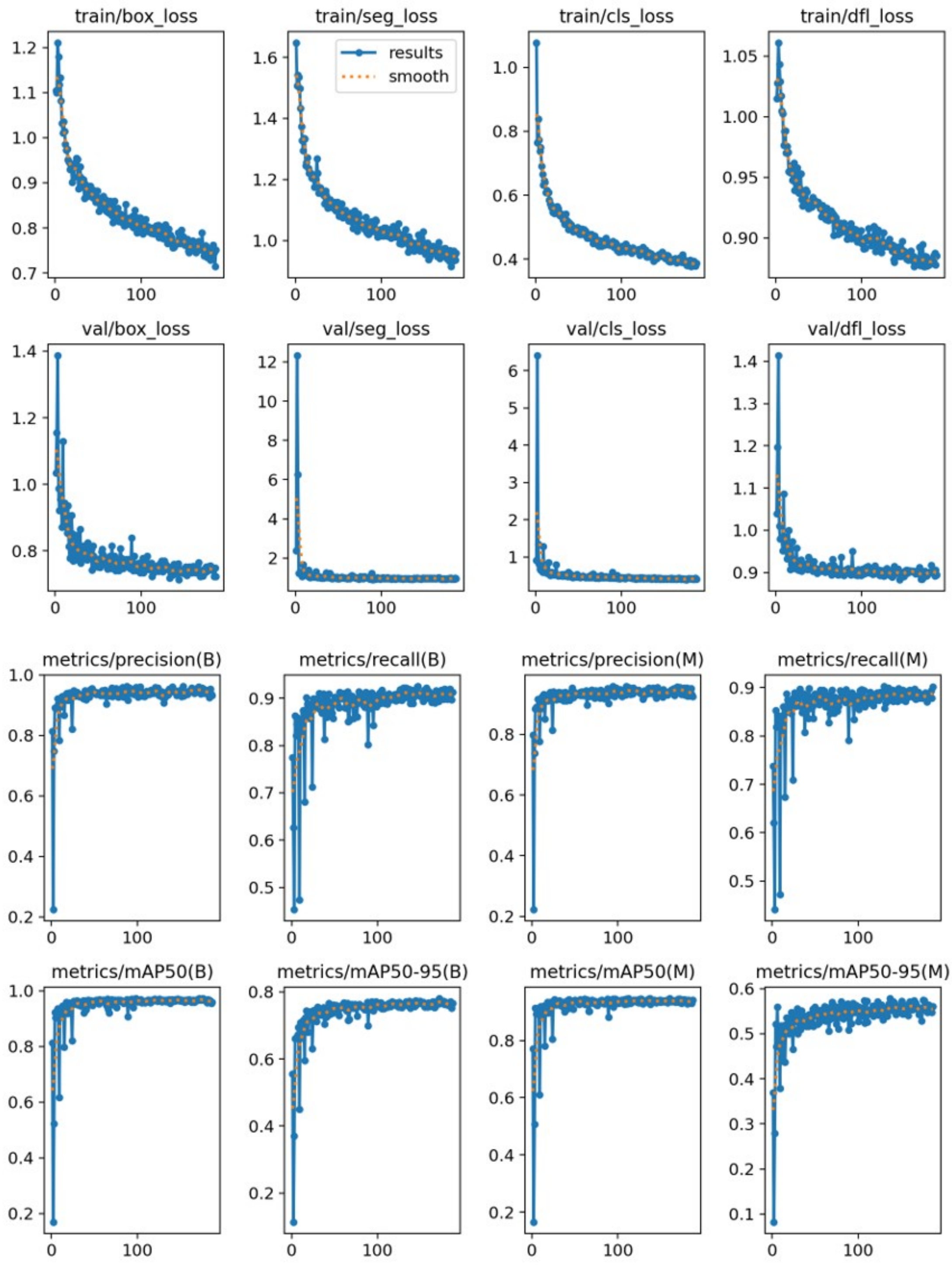

Figure S3. Changes in losses, precision, recall, mAP50 and mAP50-95 over epochs during model training.

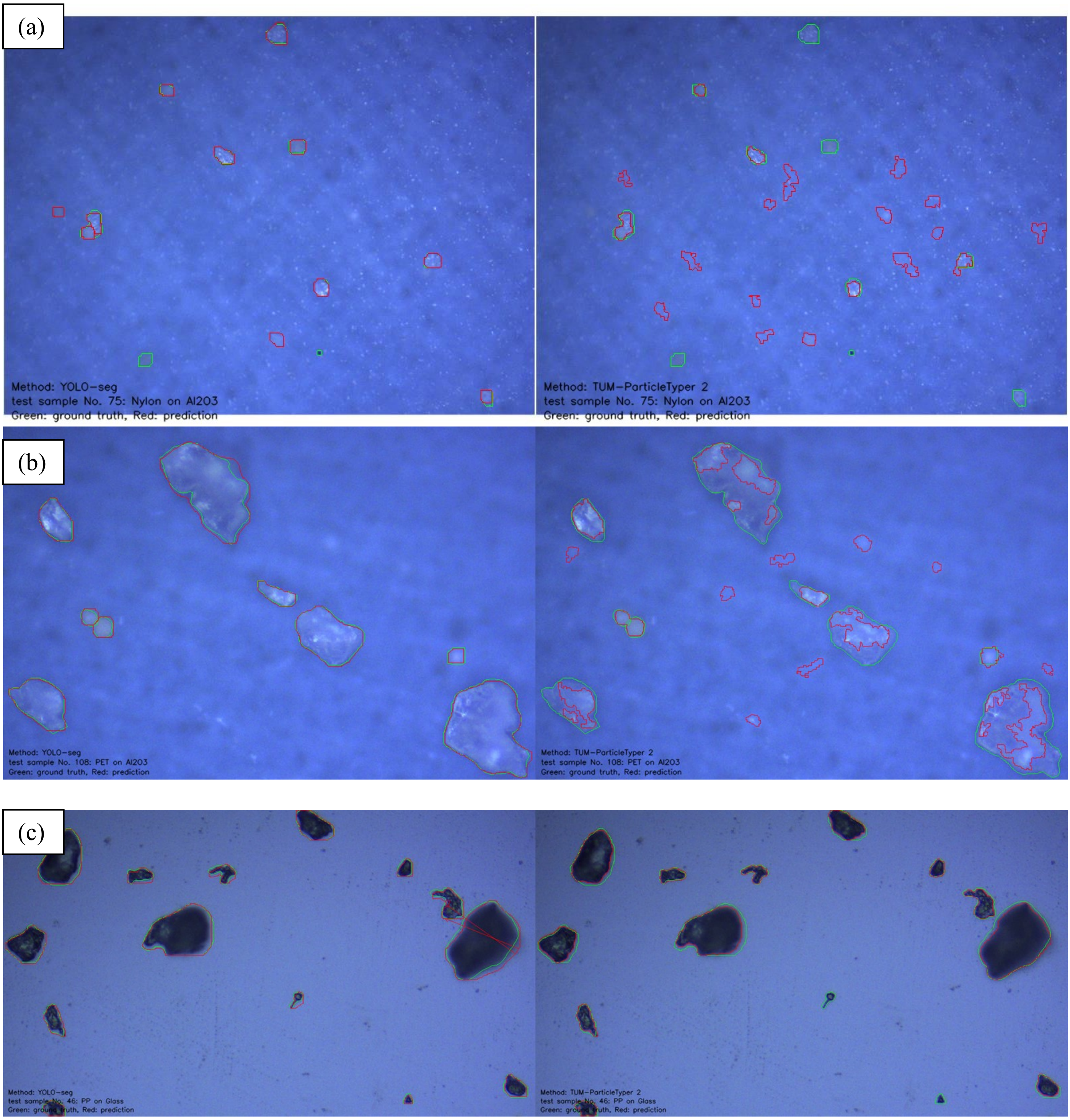

Figure S4. Segmentation results generated by the YOLO-seg model developed in this study and by TUM-ParticleTyper 2, showing only the contours of the segmented regions, overlaid with ground truth contours. Green: ground truth, Red: segmentation generated by YOLO-seg or TUM-ParticleTyper 2. Results are presented for a test image of Nylon particles on Al_2O_3 filter (a), a test image of PET particles an Al_2O_3 filter (b), and a test image of PP particles on glass slide (c). Relevant information is provided in the lower-left corner of each image. Results

on three test images are shown in the Supplementary Material; the segmentation results on all 63 test images are available at the following link in the folder "Segmentation results". https://drive.google.com/drive/folders/1g3lnhXggQYbZrCQPiHk1EXdFZ2zKLQO4?usp=sharing

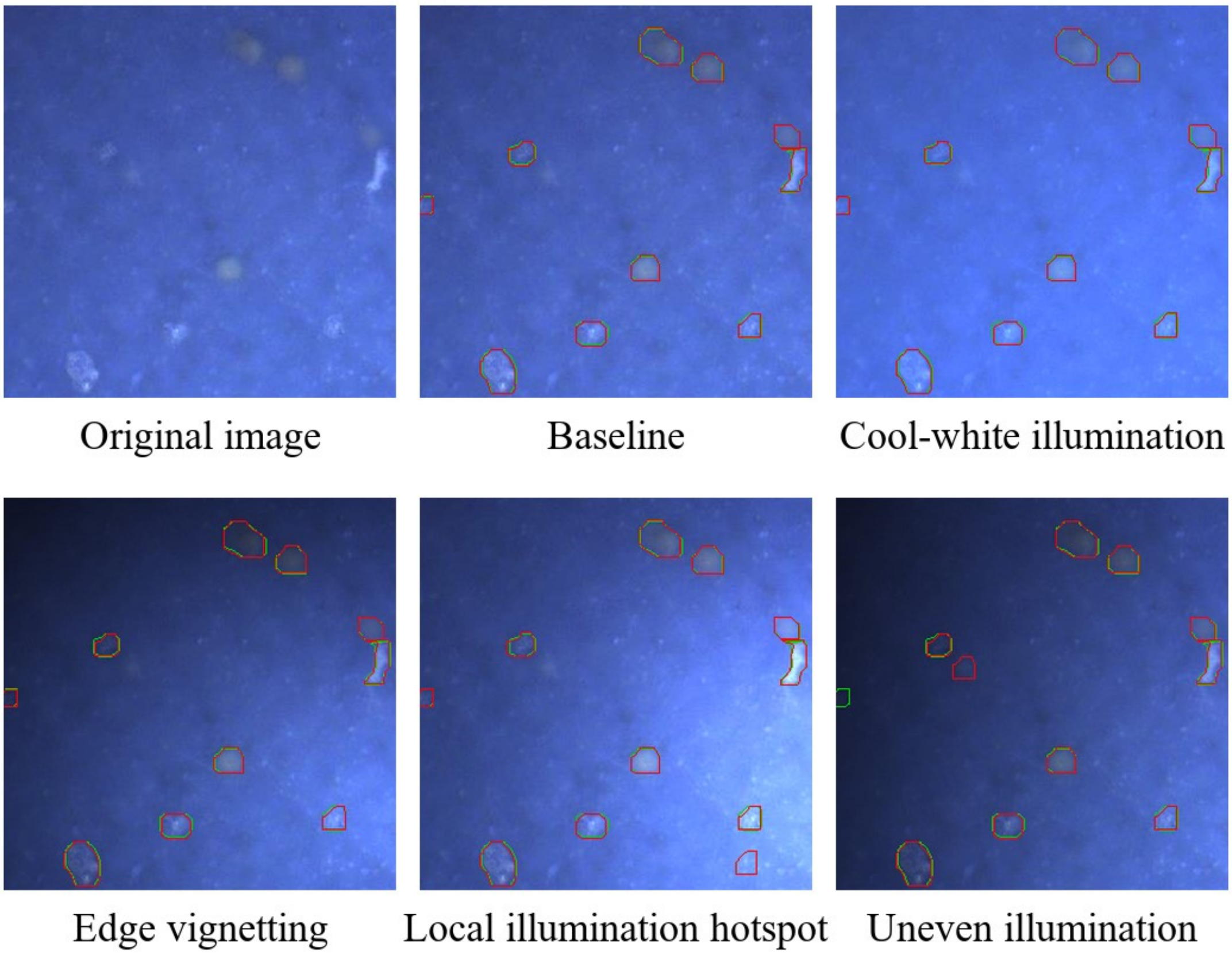

Figure S5. YOLO-seg contour comparison for the same representative image region under different illumination conditions. The first panel shows the original image region without contour overlays. The remaining panels show the baseline and illumination-perturbed results. Green contours indicate ground-truth annotations, and red contours indicate YOLO-seg predictions.

Table S1. Effect of illumination perturbations on YOLO-seg segmentation performance on the test set.

Imaging condition	mAP@0.5	Change vs. baseline	mAP@0.5:0.95	Change vs. baseline
Baseline	0.959	0.000	0.592	0.000
Cool-white	0.951	-0.008	0.587	-0.005

illumination				
Edge vignetting	0.958	-0.001	0.592	0.000
Local illumination hotspot	0.957	-0.002	0.590	-0.002
Uneven illumination	0.950	-0.009	0.581	-0.011

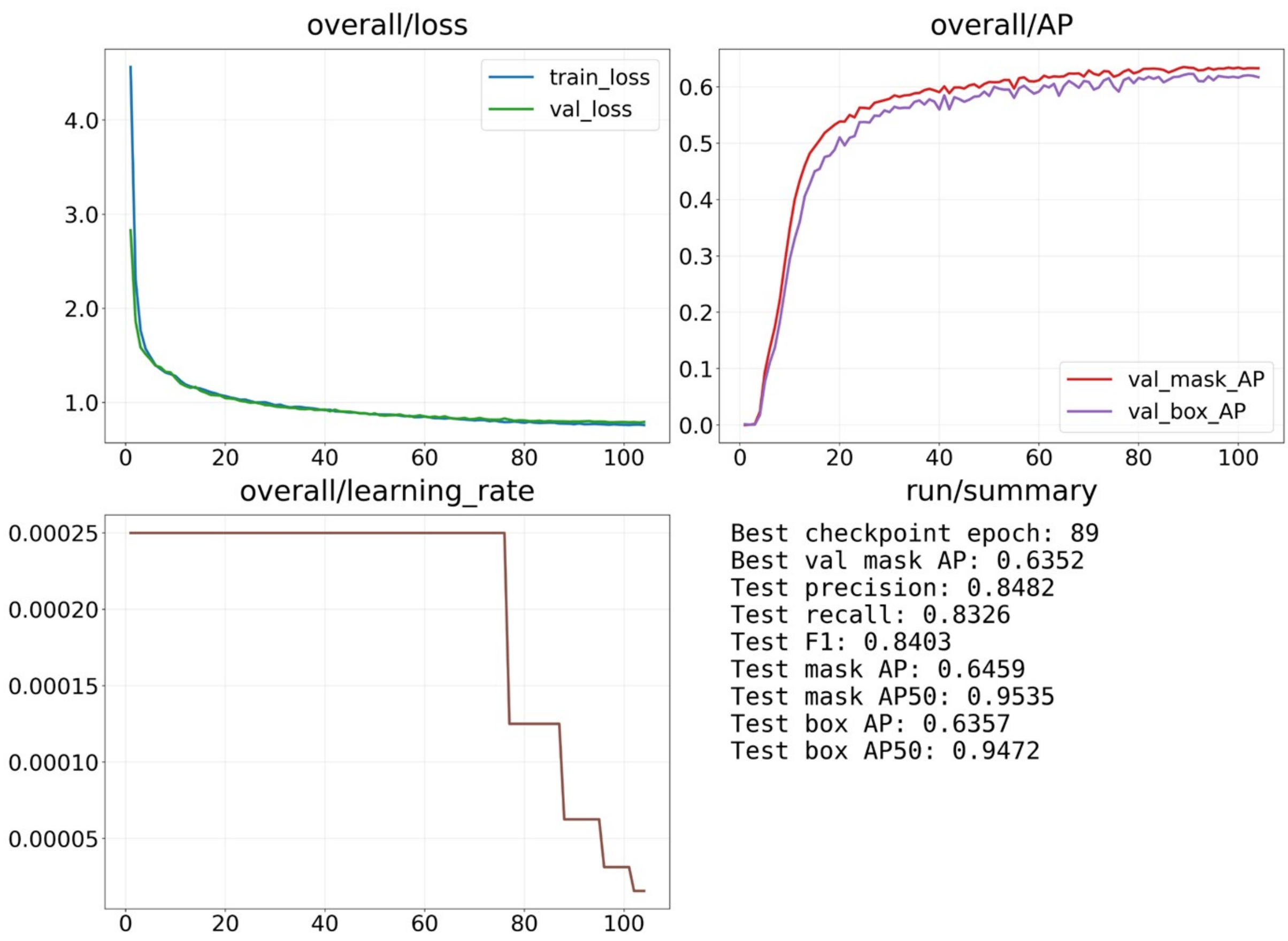

Figure S6. Training dynamics and performance summary of Mask R-CNN, including the training and validation losses, validation AP curves, learning-rate schedule, and the best-checkpoint test performance.

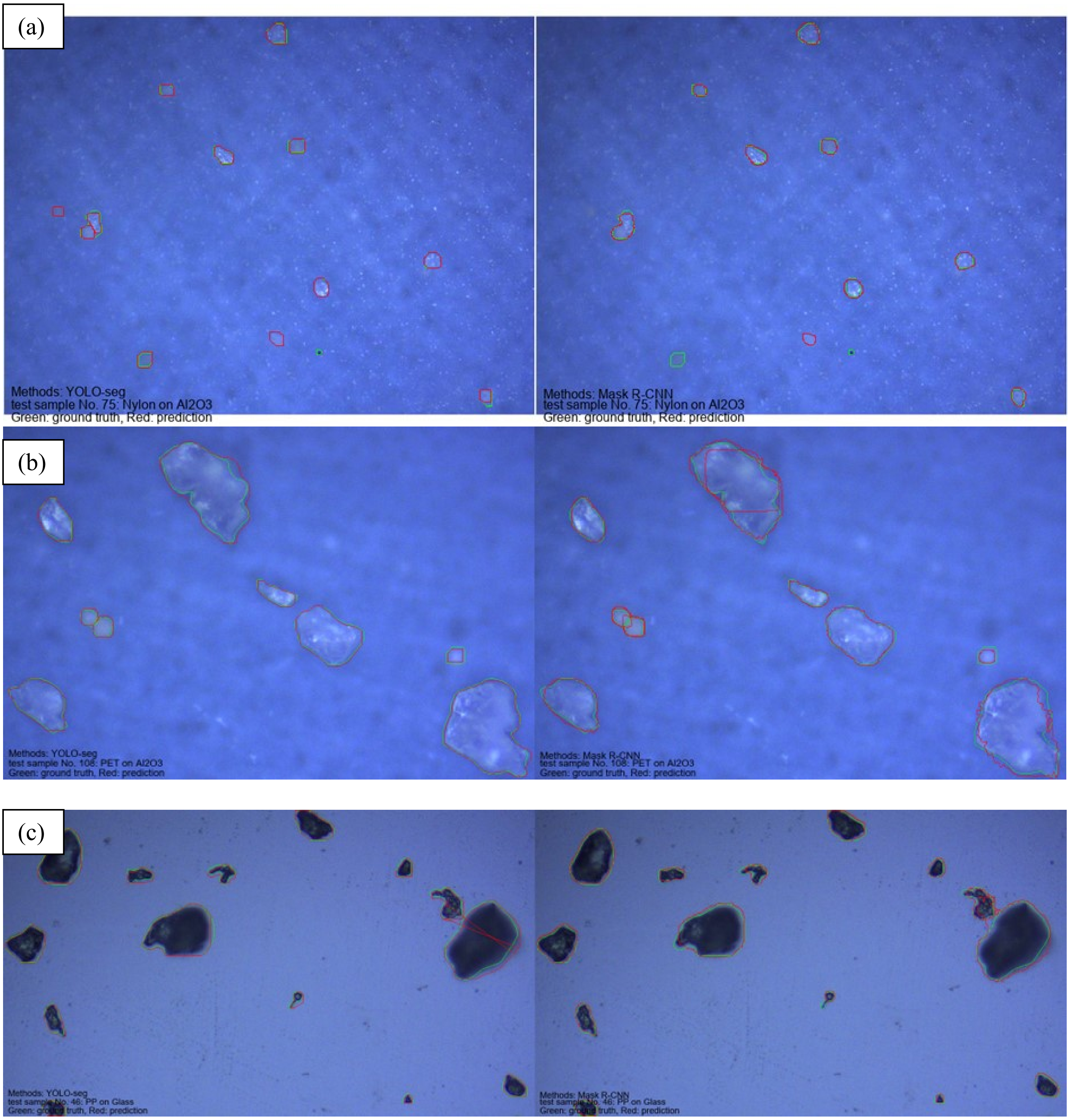

Figure S7. Segmentation results generated by the YOLO-seg model and by the Mask R-CNN model, showing only the contours of the segmented regions, overlaid with ground truth contours. Green: ground truth, Red: segmentation generated by YOLO-seg or Mask R-CNN. Results are presented for a test image of Nylon particles on Al_2O_3 filter (a), a test image of PET particles an Al_2O_3 filter (b), and a test image of PP particles on glass slide (c). Relevant information is provided in the lower-left corner of each image. Results on three test images are

shown in the Supplementary Material; the segmentation results on all 63 test images are available at the following link in the folder “Segmentation results”. https://drive.google.com/drive/folders/1g3lnhXggQYbZrCQPiHk1EXdFZ2zKLQO4?usp=sharing

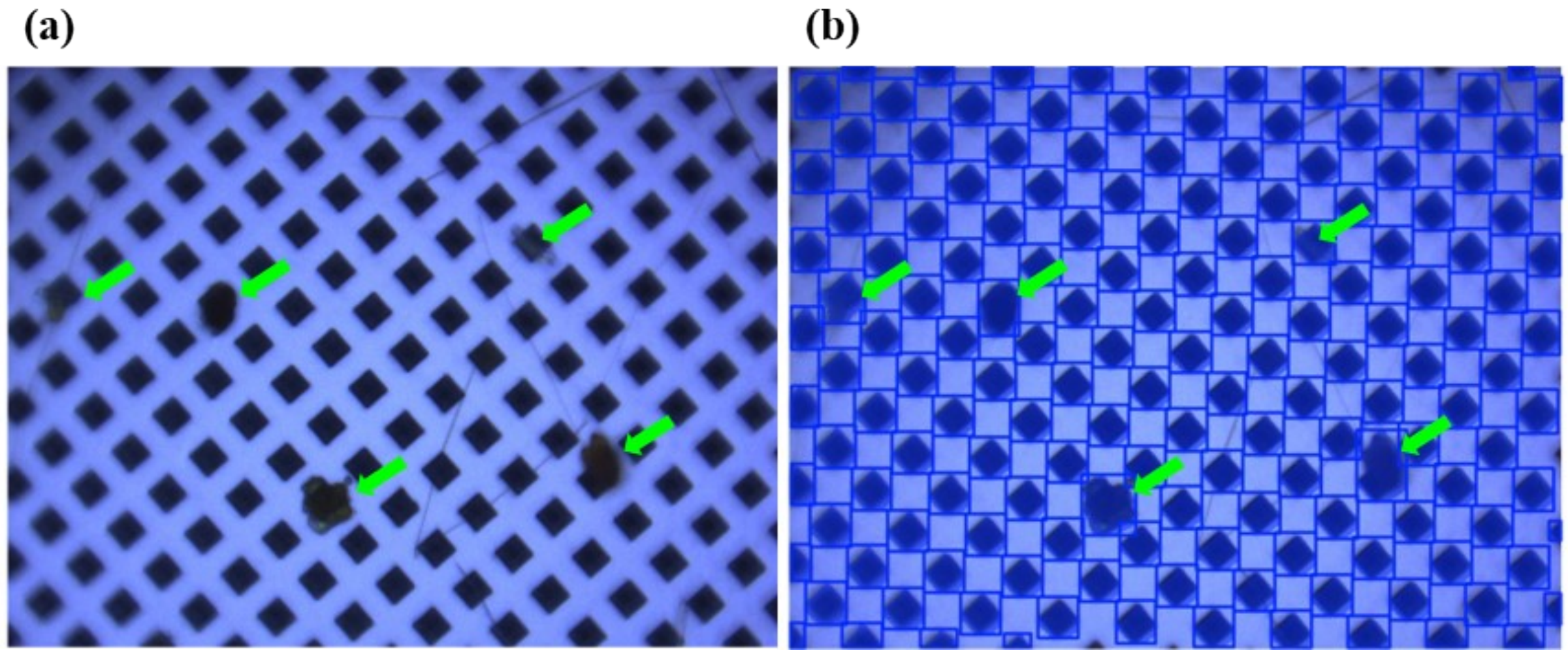

Figure S8. Optical micrograph of a 166 μm × 124 μm area of the silicone membrane used to filter tap water in this study (a) and YOLO-seg segmentation results on the same image (b). Each detected region is enclosed by its corresponding bounding box.

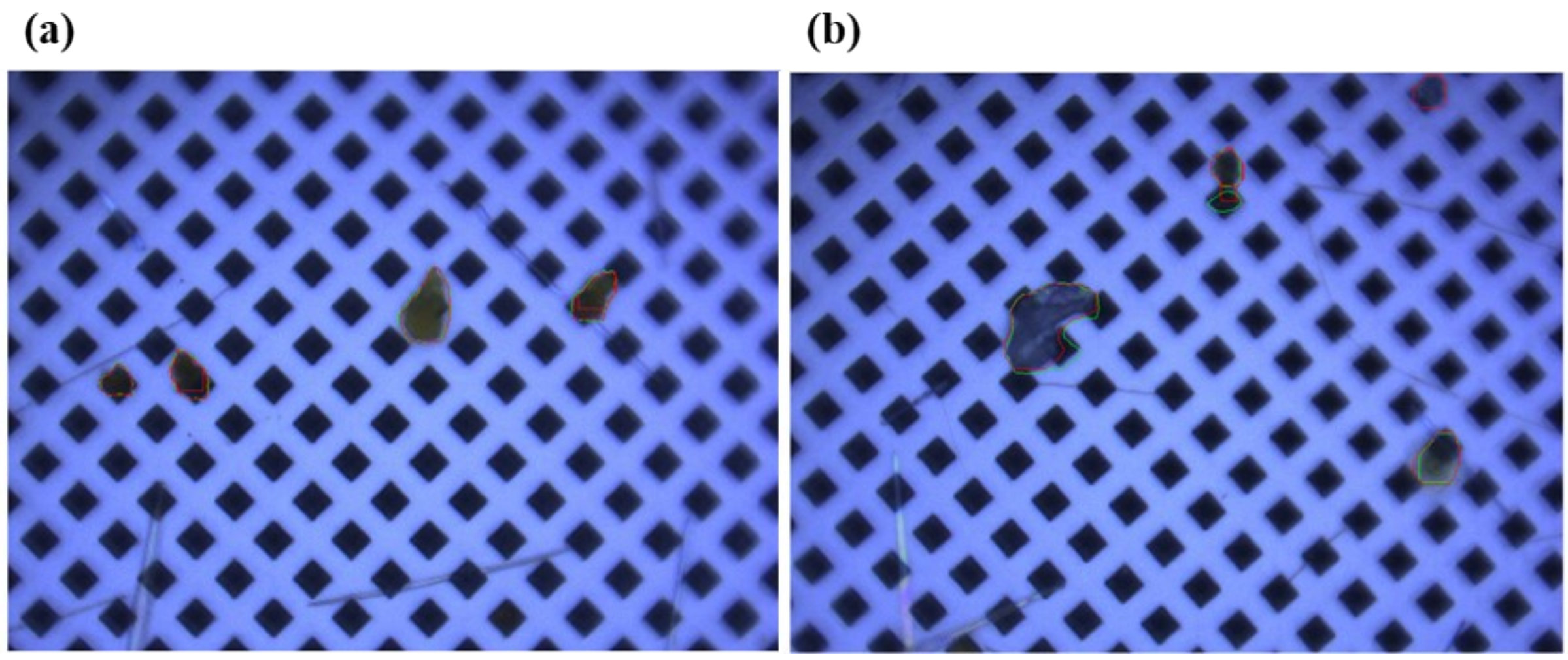

Figure S9. Segmentation results on the images of the tap water sample, generated by the YOLO-seg model, showing only the contours of the segmented regions, overlaid with ground truth contours. Green: ground truth, Red: segmentation generated by YOLO-seg. Results on two images are shown in the Supplementary Material; the segmentation results on all 1,223 images of the tap water sample are available at the following link in the folder “Segmentation results”.

https://drive.google.com/drive/folders/1g3lnhXggQYbZrCQPiHk1EXdFZ2zKLQO4?usp=sharing

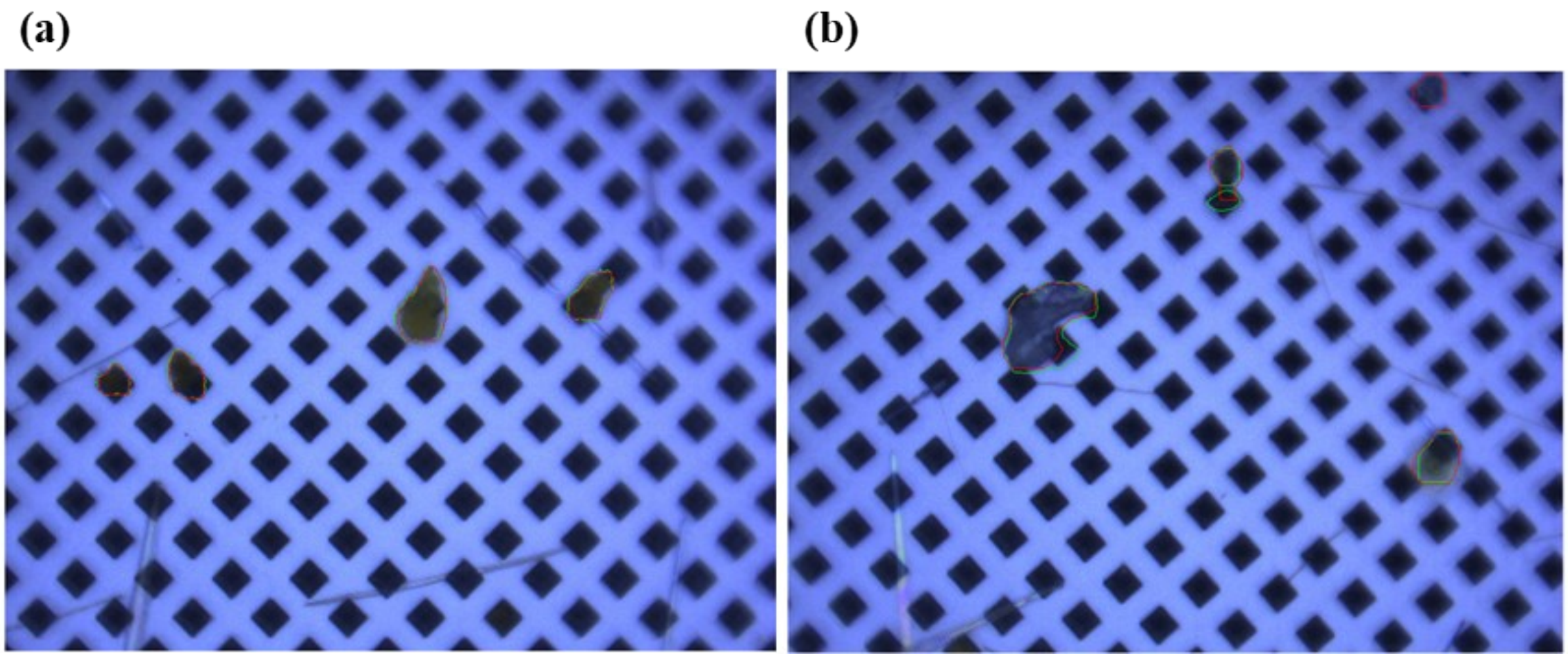

Figure S10. Segmentation results on the images of the tap water sample, the segmented regions were generated by the YOLO-seg model, then a postprocessing technique was applied to remove 346 false positives (IOU=0.7). Only the contours of the segmented regions are shown, overlaid with ground truth contours. Green: ground truth contours; Red: segmentation contours. Results on two images are shown in the Supplementary Material; the segmentation results on all 1,223 images of the tap water sample are available at the following link in the folder "Segmentation results". https://drive.google.com/drive/folders/1g3lnhXggQYbZrCQPiHk1EXdFZ2zKLQO4?usp=sharing

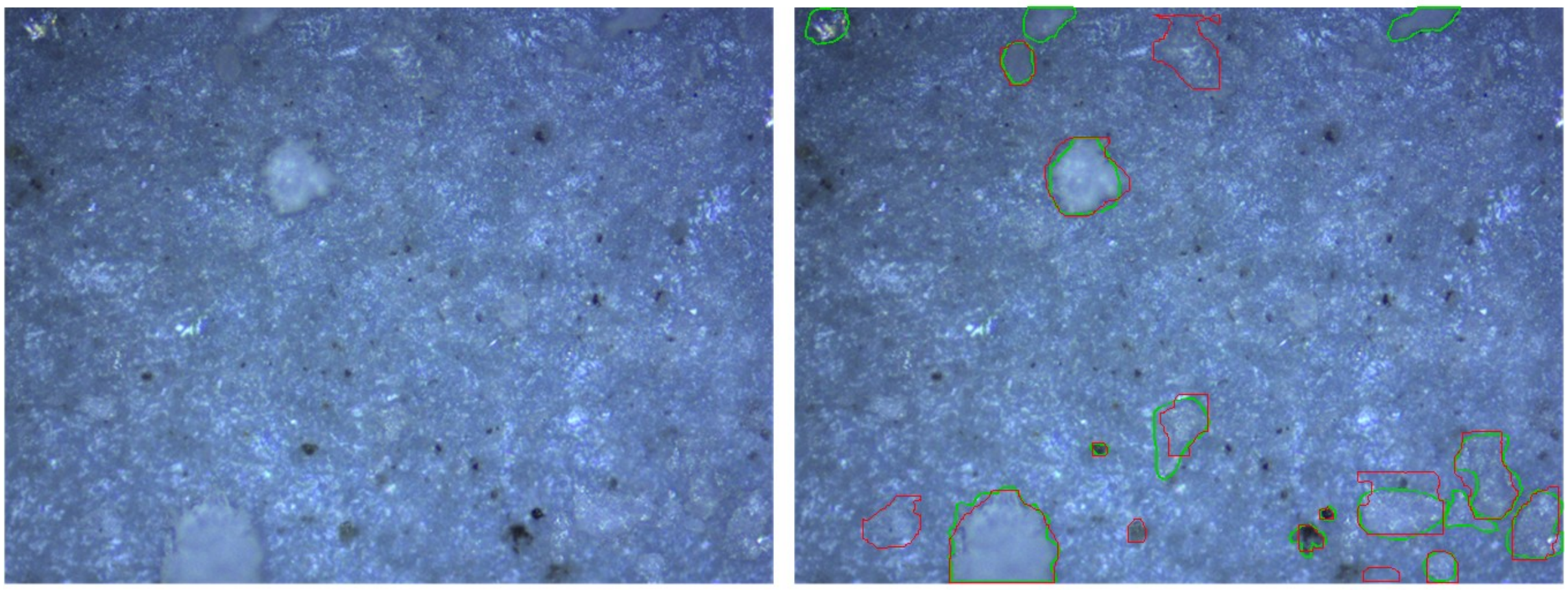

Figure S11. Segmentation contours produced by the adapted YOLO segmentation model on one example test image from the commercial salt sample deposited on an Al_2O_3 filter. The left panel shows the original optical image, and the right panel shows the same image with overlaid

contours. Green: ground truth contours; red: segmentation contours generated by the adapted YOLO segmentation model. Only one example is shown in the Supplementary Material; visualized results across all test images from the commercial salt sample are available at the following Google Drive link in the folder “Segmentation results”. https://drive.google.com/drive/folders/1g3lnhXggQYbZrCQPiHk1EXdFZ2zKLQO4?usp=sharing

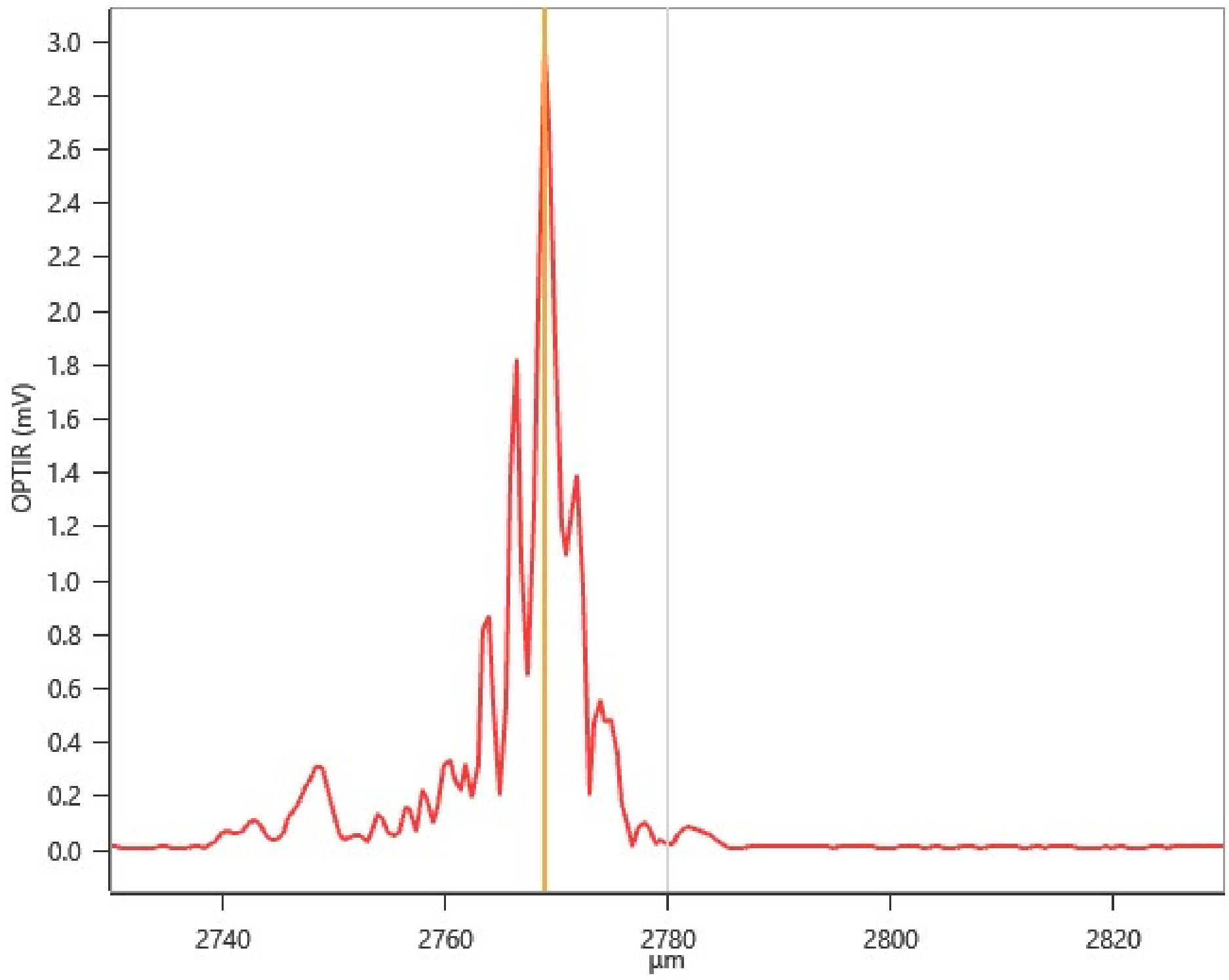

Figure S12. IR responses at 1639 cm^{-1} from a 15 µm nylon particle measured at different focal positions. OPTIR (mV) represents the optical photothermal signal, reported in millivolts, which reflects the corresponding IR response.
